# Ebastine impairs metastatic spread in triple-negative breast cancer by targeting focal adhesion kinase

**DOI:** 10.1007/s00018-023-04760-5

**Published:** 2023-04-25

**Authors:** Juyeon Seo, Minsu Park, Dongmi Ko, Seongjae Kim, Jung Min Park, Soeun Park, Kee Dal Nam, Lee Farrand, Jinsol Yang, Chaok Seok, Eunsun Jung, Yoon-Jae Kim, Ji Young Kim, Jae Hong Seo

**Affiliations:** 1 Division of Medical Oncology, Department of Internal Medicine, Korea University College of Medicine, Korea University, Seoul, 02841 Republic of Korea; 2 Brain Korea 21 Program for Biomedical Science, Korea University College of Medicine, Korea University, Seoul, 02841 Republic of Korea; 3 Department of Biomedical Research Center, Korea University Guro Hospital, Korea University, Seoul, 08308 Republic of Korea; 4 Adelaide Medical School, Faculty of Health and Medical Sciences, The University of Adelaide, Adelaide, SA 5000 Australia; 5 Galux Inc, Gwanak-Gu, Seoul, 08738 Republic of Korea; 6 Department of Chemistry, Seoul National University, Seoul, 08826 Republic of Korea; 7 Guro Hospital Campus, Korea University, 97 Gurodong-Gil, Guro-Guu, Seoul, 08308 Republic of Korea

**Keywords:** Ebastine, Triple-negative breast cancer, FAK, Breast cancer stem cells, Metastasis, Drug repurposing

## Abstract

We sought to investigate the utility of ebastine (EBA), a second-generation antihistamine with potent anti-metastatic properties, in the context of breast cancer stem cell (BCSC)-suppression in triple-negative breast cancer (TNBC). EBA binds to the tyrosine kinase domain of focal adhesion kinase (FAK), blocking phosphorylation at the Y397 and Y576/577 residues. FAK-mediated JAK2/STAT3 and MEK/ERK signaling was attenuated after EBA challenge in vitro and in vivo. EBA treatment induced apoptosis and a sharp decline in the expression of the BCSC markers ALDH1, CD44 and CD49f, suggesting that EBA targets BCSC-like cell populations while reducing tumor bulk. EBA administration significantly impeded BCSC-enriched tumor burden, angiogenesis and distant metastasis while reducing MMP-2/-9 levels in circulating blood in vivo. Our findings suggest that EBA may represent an effective therapeutic for the simultaneous targeting of JAK2/STAT3 and MEK/ERK for the treatment of molecularly heterogeneous TNBC with divergent profiles. Further investigation of EBA as an anti-metastatic agent for the treatment of TNBC is warranted.

**Supplementary Information:**

The online version contains supplementary material available at 10.1007/s00018-023-04760-5.

## Introduction

Breast cancer can be classified into four phenotypes including luminal A, luminal B, HER2-positive, and triple-negative breast cancer (TNBC), according to the molecular expression of estrogen receptor (ER), progesterone receptor (PR), and human epidermal growth factor receptor 2 (HER2) [1]. TNBC is negative for ER, PR, and HER2, and there are therefore no targeted therapies available for TNBC patients, with the standard of care relying on cytotoxic therapy [2]. According to clinical findings reported in 2021, the 5-year relative survival rate for TNBC patients with distant metastasis is only 12%, highlighting the critical unmet needs for these patients [3].

The high incidence of recurrence and metastasis in TNBC is thought to be due to the existence of breast cancer stem cells (BCSCs) within the primary bulk tumor [4]. BCSCs are characterized by high expression of the CD44 cell surface marker, low expression of CD24 (CD44^high^/CD24^low^), aldehyde dehydrogenase 1 (ALDH1) activity, and the capacity to form mammospheres with self-renewal properties [5, 6]. Epithelial-to-mesenchymal transition (EMT) leads to aggressive tumor growth and is responsible for the metastatic spread of cancer, conferring cell mobility and invasive behavior, beginning with the loss of basal cellular polarity and cell–cell adhesion [7].

Focal adhesion kinase (FAK) is a key component within the focal adhesion complex that harbors tyrosine kinase activity and participates in the acquisition of EMT, cell adhesion, migration and invasion, as well as the maintenance of BCSC-like traits [8, 9]. Of particular note, abnormal upregulation of phospho-FAK (Y397) is frequently observed in TNBC patients with higher histologic grades and Ki-67 proliferative index [10]. FAK consists of an N-terminal 4.1-erzin-radixin-moesin (FERM) domain, a central kinase domain with catalytic activity, and a focal adhesion targeting domain [11]. Autophosphorylation at Y397, located between the terminal and central kinase areas of the FERM domain, induces FAK/Src signaling, causing phosphorylation of the FAK catalytic domain at Y576/577 and consequently leading to JAK/STAT3, PI3K/Akt and MEK/ERK signaling [12]. These pro-survival signaling pathways are potential therapeutic targets in TNBC.

**Fig. 1:**
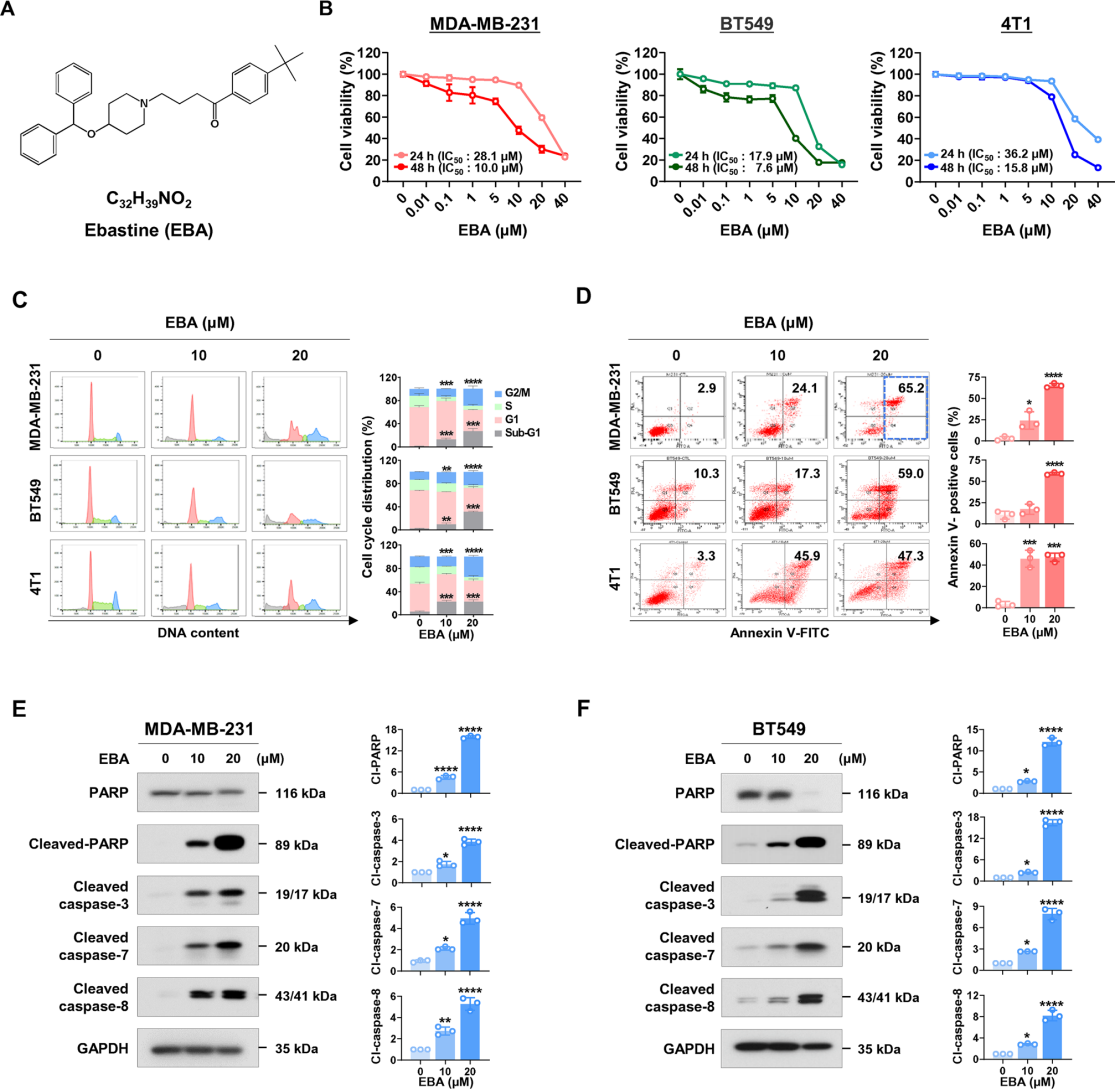
Ebastine (EBA) reduces cell viability and induces apoptosis in TNBC cells. **A** Chemical structure of ebastine (EBA). **B** MDA-MB-231, BT549 and 4T1 cells were treated with EBA (0.01–40 μM) or vehicle (DMSO) for 24 and 48 h, and cell viability and IC_50_ values were analyzed by MTS assay (n = 5). **C** Cells were treated with EBA (10–20 μM) and cell cycle analysis was achieved with flow cytometry. The proportion of cell cycle distribution was quantified by FlowJoTM v10 Software (**p < 0.01, n = 3). **D** Early and late apoptosis in cells with EBA (10–20 μM) assessed by Annexin V/PI assay (*p < 0.05, n = 3). **E–F** Effect of EBA (10–20 μM, 24 h) on PARP, cleaved-PARP, cleaved-caspase-3, -7 and -8 expression in MDA-MB-231 (**E**) and BT549 cells (**F**). GAPDH was used as an internal control. Quantitative graphs of protein contents are shown in the right panels (*p < 0.05, n = 3). The results are expressed as mean ± SD of least three independent experiments by one-way ANOVA with Bonferroni’s multiple comparison test

Ebastine (EBA) is a piperidine derivative small molecule (Fig. 1A) that exhibits antitumor effects in a variety of carcinomas [13–15]. However, the effect of EBA on TNBC stem-like properties remains unclear. Recently, several antihistamines have been reported to elicit antitumor effects in various carcinomas [14]. We sought to investigate the interaction between EBA and the FAK kinase domain in the context of anti-metastatic potential in TNBC*.*

## Results

### Ebastine (EBA) reduces cell viability and induces apoptosis in TNBC cells

TNBC cells (MDA-MB-231, BT549 and 4T1) treated with EBA (0–40 μM, 24–48 h) exhibited concentration-dependent reductions in cell viability (Fig. 1B). Cell cycle analysis revealed that EBA significantly induced G2/M phase arrest and increased sub-G1 accumulation, suggesting that EBA simultaneously induces cytostatic and cytotoxic effects in TNBC cells (*p* < 0.01, Fig. 1C). Furthermore, treatment with EBA (10–20 μM, for 48 h) resulted in markedly higher proportions of TNBC cells in early and late apoptosis (*p* < 0.05, Fig. 1D). EBA-induced apoptosis was accompanied by the activation of caspases, as evidenced by increased caspase-8 cleavage as well as caspase-3/-7 activation coinciding with subsequent PARP cleavage (*p* < 0.05, Fig. 1E–F). Treatment with EBA (0.01–40 µM, 48 h) was found to be less sensitive to normal fibroblast NIH/3T3 cells than in the TNBC cells, as evidenced by IC50 values from 2.17-fold up to 4.91-fold higher (Supplementary Fig. S1A). It is noteworthy that EBA induced enhancement of apoptosis in TNBC cells, but did not affect apoptosis in non-malignant NIH/3T3 cells (Supplementary Fig. S1B).

### EBA suppresses FAK phosphorylation by targeting its kinase domain

**Fig. 2:**
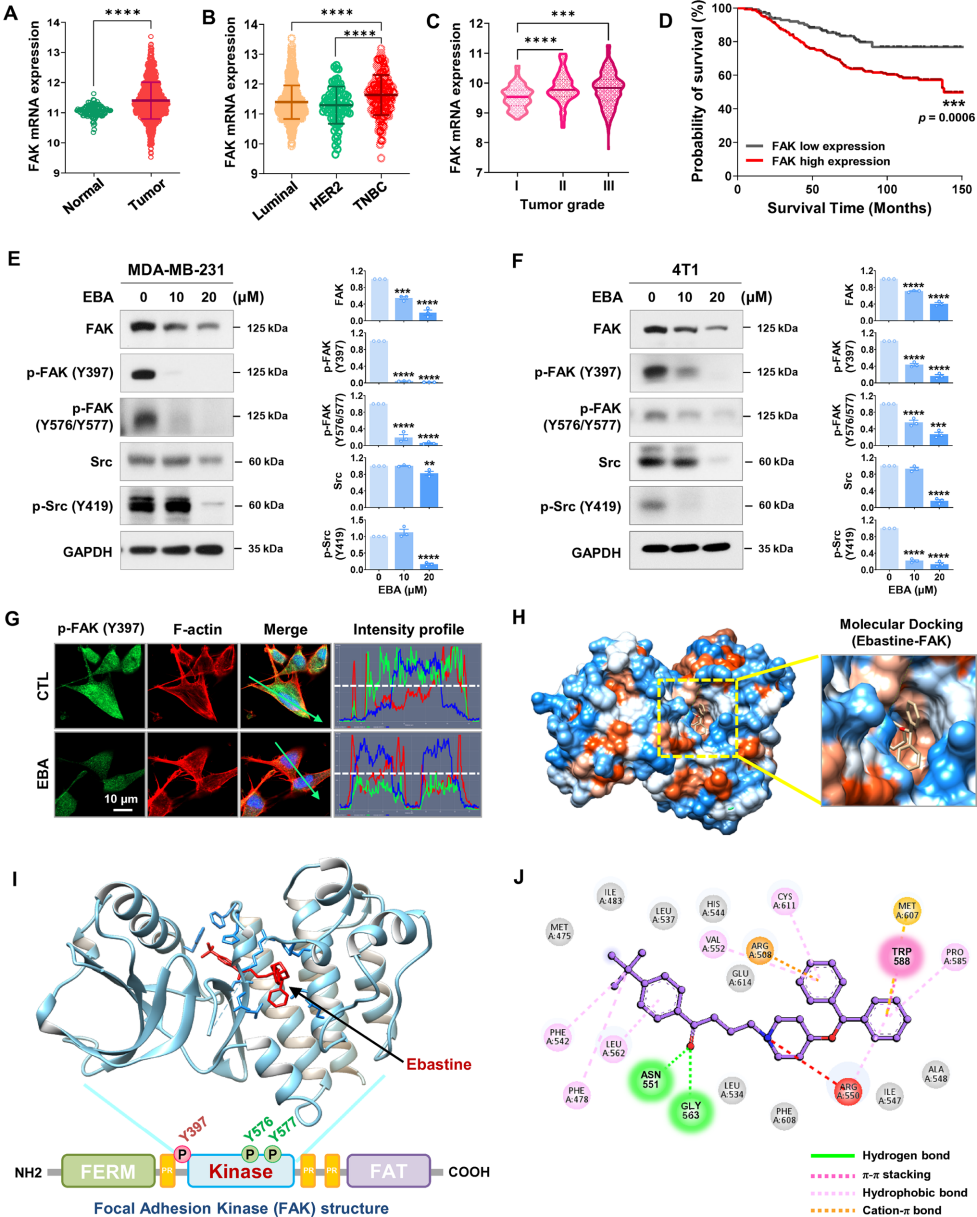
EBA impairs FAK kinase activity. **A** Analysis of mRNA expression for FAK in tumor tissues compared to normal in breast cancer patients [****p < 0.0001, Normal (n = 115) and Tumor (n = 1097)]. **B** Comparison of FAK mRNA expression in subtypes of breast cancer patients [****p < 0.0001, Luminal (n = 701), HER2-positive BC (n = 76) and TNBC (n = 165)]. **C** Differences in FAK expression according to tumor grade [***p < 0.001, I (n = 82), II (n = 193) and III (n = 450)]. **D** Comparison of Kaplan–Meier overall survival curves between FAK-high and -low groups [*log-rank; p* = *0.0006*, FAK-high (n = 270) and FAK-low (n = 114)]. **E–F** Influence of EBA (10–20 μM, 48 h) on FAK, p-FAK (Y397), p-FAK (Y576/577), Src and phospho-Src (Y419) protein content in MDA-MB-231 (**E**) and 4T1 (**F**) cells. Quantitative graphs represent the ratio of protein/GAPDH in the presence or absence of EBA (**p < 0.01, n = 3). **G** Immunofluorescence analysis of p-FAK (Y397, green) and F-actin (red) with DAPI (blue) in BT549 cells after treatment with EBA (10 μM, 48 h). The intensity profiles represent p-FAK with green signal fluorescence. **H-I** Structural docking simulation between EBA and FAK (PDB: 4EBV). **H** Lipophilicity property surface map (red: hydrophobic, blue: hydrophilic) of the active site. **I** Binding pose of EBA in the tyrosine kinase domain of FAK. The chain of the FAK kinase domain is rendered as a blue ribbon, and EBA is depicted as a red stick model. **J** 2D representation of intermolecular interactions between EBA and FAK. Key amino acid residues within the binding pocket are displayed in ball-and-stick format. Hydrogen bonding (< 5.0 Å), cation-π interactions, and π-π stacking are represented as green, yellow, and dark pink dashed lines, respectively

Analysis of the breast cancer patient dataset revealed that FAK is overexpressed in subtypes, with the highest levels in TNBC (*p* < 0.0001, Fig. 2A–B). Increases in FAK expression correlated with higher breast cancer tumor grade, and aberrant FAK overexpression was associated with reduced overall survival (*p* < 0.001, Fig. 2C–D). Exposure of MDA-MB-231, 4T1 and BT549 cells to EBA (10–20 μM, 48 h) significantly downregulated phospho-FAK (Y397, Y576/577) and phospho-Src (Y419) (*p* < 0.01, Fig. 2E–F and Supplementary Fig. S2 and S3). Confocal microscopy analysis further supported the notion that EBA treatment resulted in a marked reduction in phospho-FAK (Y397) expression in TNBC cells (Fig. 2G and Supplementary Fig. S4).

Docking simulations using the crystal structure of FAK (PDB: 4EBV, residues 410–686) showed that EBA can fit into the tyrosine kinase domain at the ATP-binding pocket between the N- and C-terminal lobes (Fig. 2H). The binding pose of EBA to FAK consists mainly of the seven hydrophobic interactions with amino acids Phe478, Phe542, Val552, Leu562, Pro585 and Cys611. Importantly, EBA is stabilized by two hydrogen bonds with the 4-ter-butylbenzonyl group, where the carboxyl oxygen atom interacts with Asn551 (3.00 Å) and Gly563 (2.75 Å). In addition, two π–stacking interactions were formed between a benzene ring in the diphenyl-methyl moiety and an active residue Trp588 (Fig. 2I–J).

### EBA targets breast cancer stem cell (BCSC)-like properties in TNBC

**Fig. 3:**
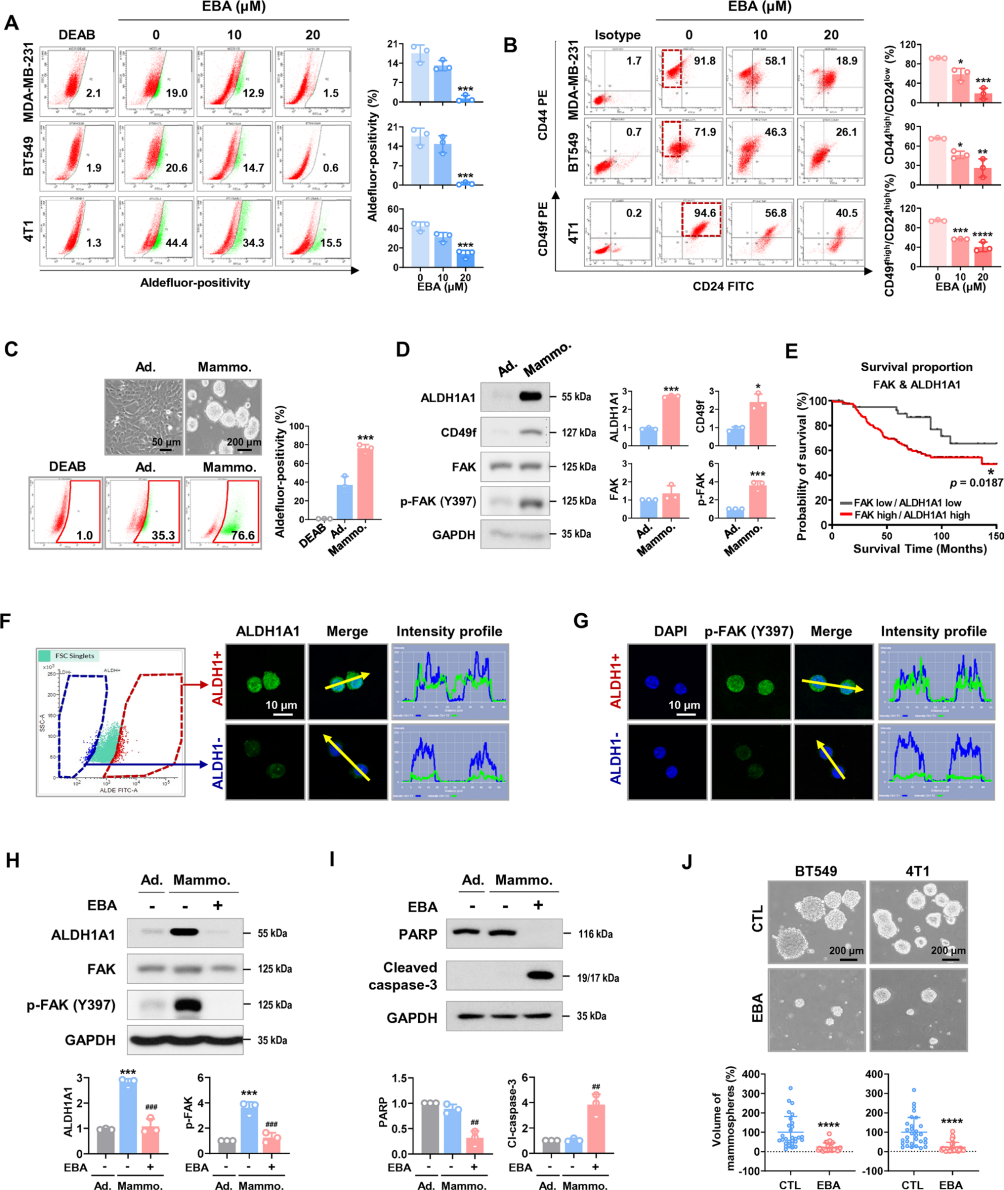
EBA targets BCSC-like properties. **A-B** MDA-MB-231, BT549 and 4T1 cells were treated with EBA (10–20 µM, 48 h). **A** Effect of EBA on ALDH1 activity (***p < 0.001, n = 3). **B** CD44^high^/CD24^low^ and CD49f^high^/CD24^high^ populations evaluated by flow cytometry. The quantitative graph represents the percentage of CD44^high^/CD24^low^ and CD49f^high^/CD24^high^ populations, respectively (*p < 0.05, n = 3). **C-D** 4T1 cells (3 × 10^4^ cells/mL) were cultured in normal culture medium or anchorage-independent serum-free 3D spheroid medium for 4 days. **C** Representative phase-contrast images and ALDH1 activity comparison between adherent 4T1 cells (Ad.) and mammospheres (Mammo.) (***p < 0.001, n = 3). **D** Comparison of expression levels of ALDH1A1, CD49f, FAK and p-FAK (Y397) between adherent cells and mammosphere groups (*p < 0.05, Ad. vs Mammo. n = 3). **E** Kaplan–Meier curves using the GENT2 database depict the overall survival of breast cancer patients with high and low FAK and ALDH1A1 expression [*log-rank*; *p* = 0.0006, FAK-low/ALDH1-low (n = 40) and FAK-high/ALDH1–high (n = 126)]. **F-G** ALDH1-positive ( +) or ALDH1-negative (-) cells were sorted from dissociated 4T1 mammospheres and immunostained for ALDH1A1 (**F**, green) and p-FAK (**G**, green) with DAPI (blue) (n = 3). Fluorescence intensity was analyzed using the intensity profiling tool in the confocal microscopy software. **H-I** Changes in the expression of ALDH1A1, FAK, p-FAK (Y397), PARP, and cleaved-caspase-3 following exposure to EBA (5 μM) for 4 days in 4T1 mammosphere culture (***p < 0.001, Ad. vs Mammo.; ##*p* < 0.01, DMSO control vs EBA treatment in mammospheres, n = 3). **J** Influence of EBA on mammosphere formation in vitro. BT549 (1 × 10^5^ cells/mL) and 4T1 cells (3 × 10^4^ cells/mL) were plated in ultralow attachment dishes, cultured in the presence or absence of EBA (5 μM) for 4 days, and the volume of mammospheres was quantified by optical microscopy (****p < 0.0001, n = 3). The results are presented as mean ± SD of at least three independent experiments and analyzed by Student’s t test or one-way ANOVA followed by Bonferroni’s multiple comparison test

Major hallmarks associated with BCSCs include aldehyde dehydrogenase 1 (ALDH1) activity, subpopulations of CD44^high^/CD24^low^ and CD49f^high^/CD24^high^, and mammosphere-forming ability [16]. An Aldefluor-positivity assay revealed concentration-dependent reductions in ALDH1 activity in response to EBA treatment (*p* < 0.001, Fig. 3A). A significant reduction in the CD44^high^/CD24^low^ stem-like population was observed in MDA-MB-231 and BT549 cells in the presence of EBA, as well as the murine BCSC-phenotype CD49f^high^/CD24^high^ population (*p* < 0.05, Fig. 3B).

Mammosphere formation can be used to analyze undifferentiated stem/progenitor cells and subpopulations with self-renewal capacity in breast cancer [17]. Mammospheres derived from 4T1 and BT549 cells had higher ALDH1A1 and CD49f protein content compared to their adherent counterparts (*p* < 0.05, Fig. 3C–D and Supplementary Fig. S5), supporting previous findings [18]. A marked upregulation of phospho-FAK (Y397) was observed during mammosphere formation (Fig. 3D). Kaplan–Meier curves revealed that the overall survival of breast cancer patients with the high expression of ALDH1A1 and FAK, shorter survival was observed (*log-rank test, p* = 0.0187, Fig. 3E). ALDH1-positive and ALDH1–negative cells were sorted from BCSC-enriched mammospheres (Fig. 3F and Supplementary Fig. S6), and fluorescence intensity analysis showed that ALDH1-positive cells had higher levels of phospho-FAK (Fig. 3G). Increased levels of the phospho-FAK and BCSC markers ALDH1A1, CD49f and Nanog in mammospheres were significantly downregulated following EBA treatment (Fig. 3H and Supplementary Fig. S7). EBA appears to eradicate BCSC-like populations, as evidenced by increased levels of cleaved caspase-3 and PARP degradation in mammospheres (*p* < 0.01, Fig. 3I). This response correlated with significant impairment of mammosphere formation in TNBC cells by EBA treatment (*p* < 0.001, Fig. 3J and Supplementary Fig. S8 and S9).

### EBA simultaneously targets multiple pro-survival signaling pathways

**Fig. 4:**
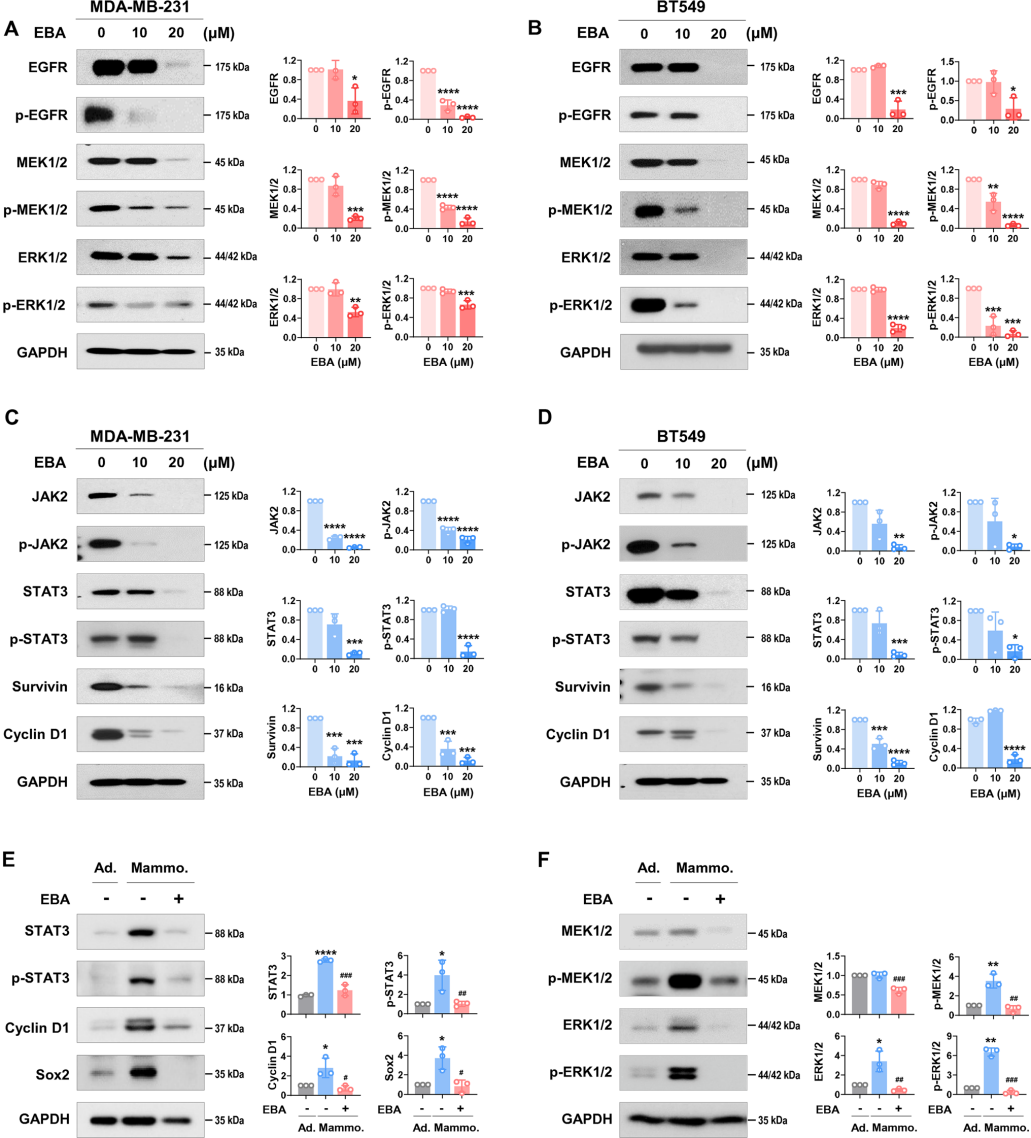
EBA downregulates EGFR and JAK2/STAT3 signaling. **A-B** Changes in the expression of EGFR, p-EGFR (Y1068), MEK1/2, p-MEK1/2 (Ser217/221), ERK1/2 and p-ERK1/2 (T202/Y204) following exposure to EBA (0–20 μM, 48 h) in MDA-MB-231 (**A**) and BT549 cells (**B**) (*p < 0.05, n = 3). **C-D** Immunoblot analyses of JAK2, p-JAK2 (Y1007/ Y 1008), STAT3, p-STAT3 (Y705), survivin and cyclin D1 in MDA-MB-231 (**C**) and BT549 cells (**D**) after treatment with EBA (0–20 μM, 48 h). Quantitative graphs represent the ratio of protein/GAPDH in the presence or absence of EBA (*p < 0.05, n = 3). **E–F** Effect of EBA on the expression of STAT3, p-STAT3 (Y705), cyclin D1, Sox2, MEK1/2, p-MEK1/2 (S217/221), ERK1/2 and p-ERK1/2 (T202/Y204) in 4T1 mammospheres. 4T1 cells (3 × 10^4^ cells/mL) were cultured in normal culture medium or serum-free suspension conditions in the presence or absence of EBA (5 μM) for 4 days (*p < 0.05, Ad. vs Mammo.; #*p* < 0.05, DMSO control vs EBA treatment in mammospheres, n = 3). The results are presented as mean values ± SD of at least three independent experiments and analyzed by Student’s t test or one-way ANOVA followed by Bonferroni’s multiple comparison test

EBA significantly downregulated protein levels of EGFR, MEK, and ERK and suppressed their phosphorylation in MDA-MB-231 and BT549 cells (*p* < 0.05, Fig. 4A–B and Supplementary Fig. S10A-B). Marked reductions in the expression and phosphorylation of JAK2/STAT3 after EBA treatment was followed by downregulation of the downstream factors cyclin D1 and survivin (*p* < 0.05, Fig. 4C–D and Supplementary Fig. S10C-D). MEK/ERK and STAT3 signaling was highly activated in BCSC-enriched 4T1 mammospheres, but these effects were completely abolished following EBA challenge (*p < 0.05, #*p* < 0.05, Fig. 4E-F).

### EBA suppresses BCSC-enriched tumor growth

**Fig. 5:**
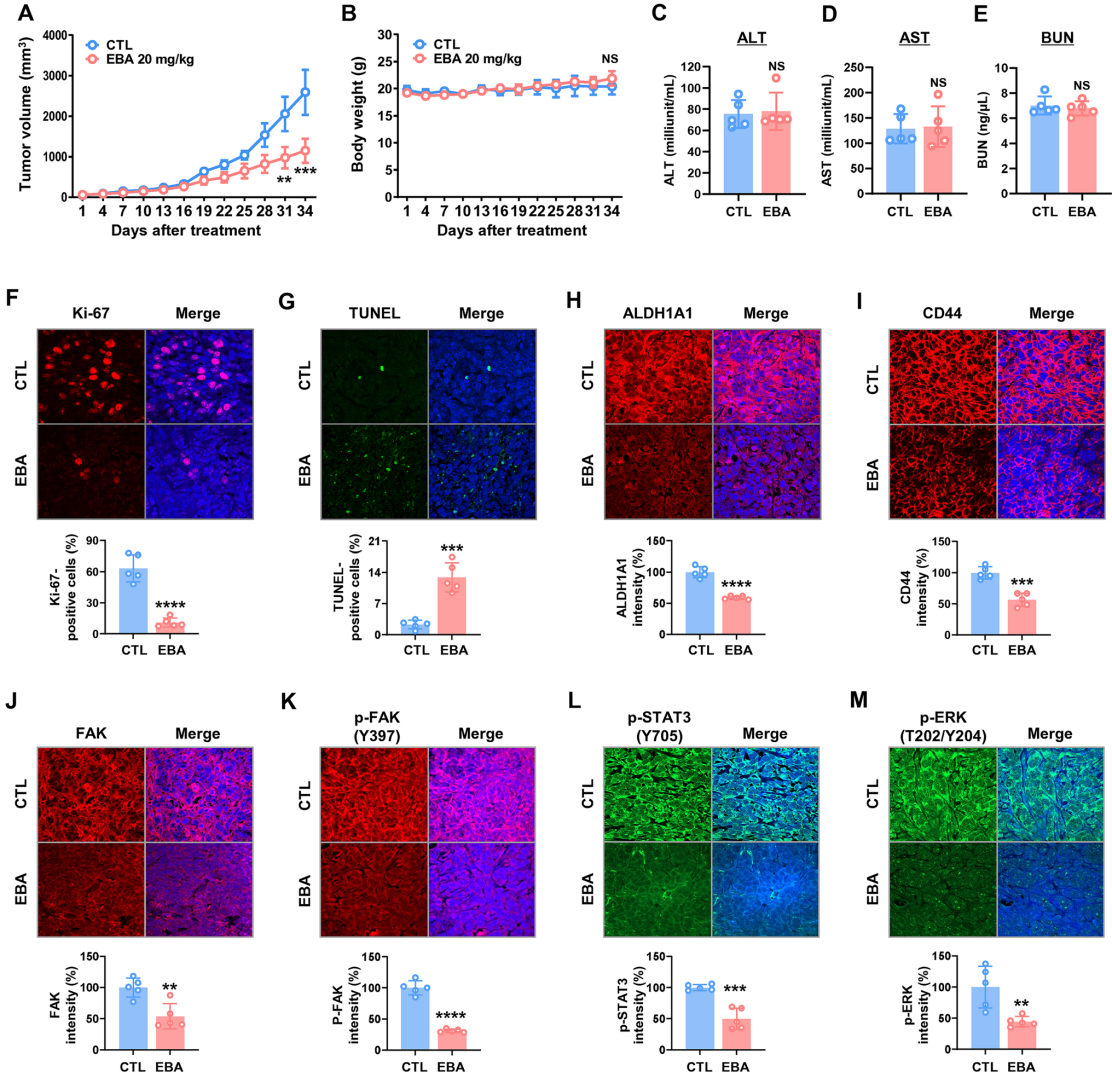
EBA inhibits tumor growth in BCSC-enriched 4T1 allografts. **A-B** Dissociated cells (1 × 10^5^) from 4T1 mammospheres were injected into the fourth mammary fat pads of BALB/c female mice. Following exposure to EBA (20 mg/kg, every other day) or solvent control for 34 days (n = 5/each group), tumor growth (**A**, **p < 0.01) and body weight (**B**, NS, not significant) were evaluated. **C-E** Effects of EBA on serum biochemical parameters of liver and kidney function (NS, not significant; ALT, alanine aminotransferase; AST, aspartate aminotransferase; BUN, blood urea nitrogen, n = 5). **F-G** Impact of EBA on Ki-67 expression and apoptosis in vivo. Tissue sections were immunostained for Ki-67 (red) with DAPI (blue), and Ki-67-positive cells were counted (**F**, ****p < 0.0001, n = 5). EBA induces apoptosis in allograft tumors, as determined by TUNEL assay. The percentage of TUNEL-positive cells was counted (**G**, ***p < 0.001, n = 5). **H-I** Influence of EBA on the expression of BCSC markers in vivo. The fluorescence intensities of ALDH1A1 (**H**, ****p < 0.0001, n = 5) and CD44 (**I**, ***p < 0.001, n = 5) were quantified. **J-M** Immunohistochemical analysis for FAK, p-FAK (Y397), p-STAT3 (Y705) and p-ERK (T202/Y204) expression in vivo. Tumor tissue sections were immunostained for FAK (**J**, red), p-FAK (**K**, red), p-STAT3 (**L**, green) or p-ERK (**M**, green) with DAPI (blue). Quantitative graphs of signal intensities are shown in the bottom panels (**p < 0.01, n = 5). All images were taken with a confocal microscope (original magnification: × 500). The fluorescence intensities were analyzed using a histogram tool in the Carl Zeiss software package. Data were analyzed by Student’s t-test or two-way ANOVA followed by Bonferroni’s multiple comparison test

EBA administration led to a significant reduction in the growth (*p* < 0.01, Fig. 5A) of BCSC-enriched 4T1 mammospheres orthotopically injected into the mammary glands of Balb/c mice. There were no significant differences in the markers of hepatic and renal impairment, ALT (NS, Fig. 5C), AST (NS, Fig. 5D) and BUN (NS, Fig. 5E), in blood serum.

The antitumor effects of EBA were accompanied by a significant decrease in Ki-67 expression (*p* < 0.0001, Fig. 5F and Supplementary Fig. S11A) and increased TUNEL-positivity (*p* < 0.001, Fig. 5G and Supplementary Fig. S11B). It was further confirmed that expression of ALDH1A1, CD44 (*p* < 0.001, Fig. 5H–Iand Supplementary Fig. S12) and CD49f (*p* < 0.0001, and Supplementary Fig. S13) were markedly attenuated in response to EBA administration in vivo. Animals receiving EBA exhibited significantly lower levels of FAK and phospho-FAK when compared to their control counterparts (*p* < 0.01, Fig. 5J–K and Supplementary Fig. S14). Furthermore, EBA exposure impaired phosphorylation of STAT3 (Y705) and ERK (T202/Y204) in the BCSC-enriched tumors (*p* < 0.01, Fig. 5L–M and Supplementary Fig. S15).

### EBA inhibits TNBC metastasis and angiogenesis via dysregulation of MMPs

**Fig. 6:**
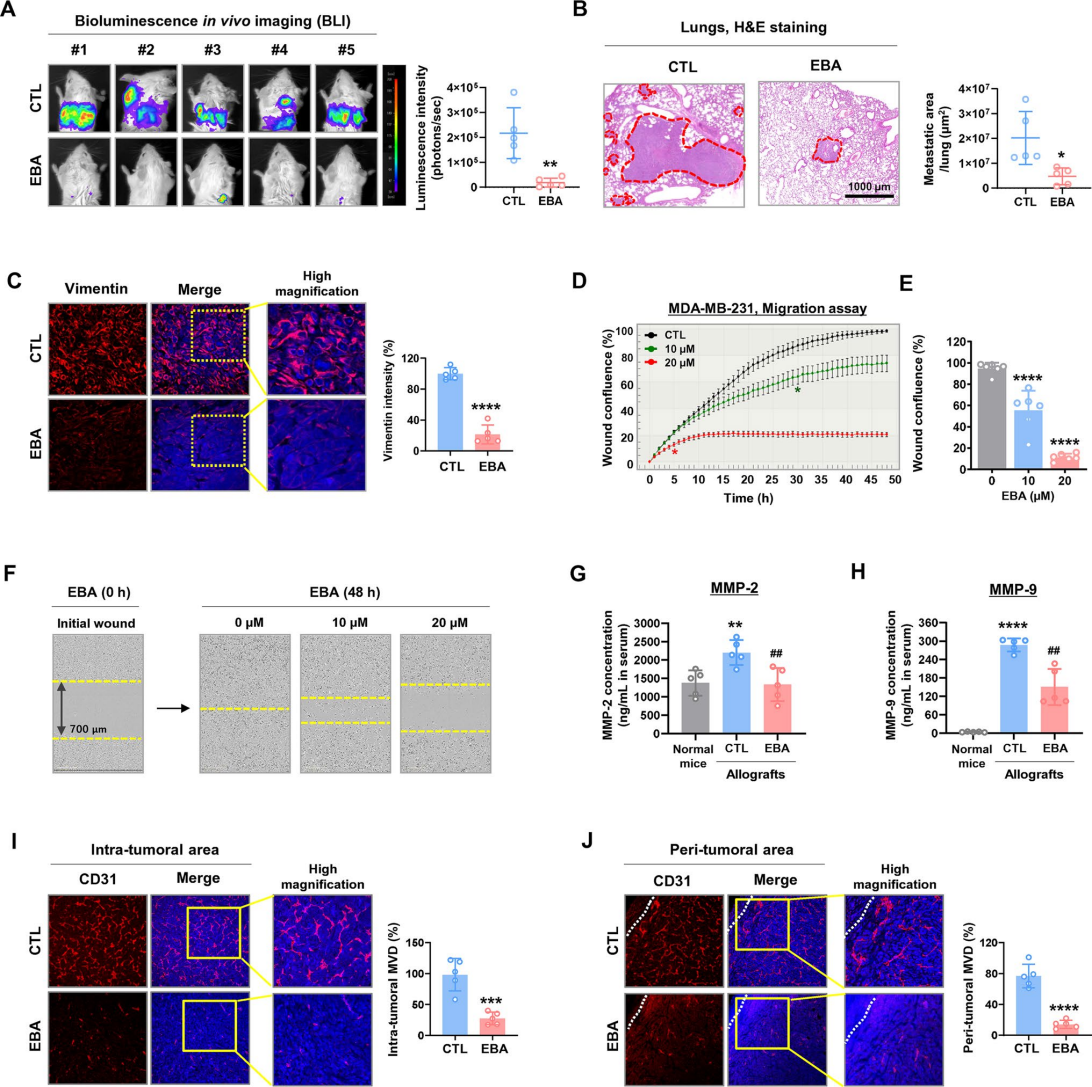
EBA suppresses TNBC metastasis. **A** Representative bioluminescence imaging (BLI) of metastasis in allografts derived from 4T1 mammospheres in vivo*.* EBA treatment caused a significant decrease in bioluminescence signal intensity (**p < 0.01, n = 5). **B** H&E staining analysis of lung sections from control and EBA-treated mice. The red dotted areas indicate metastatic lesions in the lungs (*p < 0.05, n = 5). **C** EBA administration resulted in a significant reduction in vimentin expression (****p < 0.0001, n = 5). **D-F** Effect of EBA on TNBC cell migration in vitro. **D** After treatment with EBA (0–20 µM) in MDA-MB-231 cells, kinetic analysis of cell migration was determined using the IncuCyte™ Live-Cell Imaging System. **E** Relative wound density (%) in MDA-MB-231 cells at 48 h (****p < 0.0001, n = 6). **F** Representative images of wound closure by cell migration at 0 and 48 h after EBA treatment (0–20 μM). The yellow dotted line indicates the edge of the scratched wound. **G-H** Impact of EBA on serum levels of MMP-2 and MMP-9 in vivo. MMP-2 (**G**) and MMP-9 (**H**) expression in serum extracted from CTL and EBA-treated mice determined by ELISA. Serum from normal mice was used as a negative control (**p < 0.01, normal mouse vs control allografts; ##*p* < 0.01, control allografts vs EBA-treated allografts, n = 5). **I-J** Effect of EBA on angiogenesis, as determined by a microvessel density (MVD) assay. Tumor tissues were immunostained with the specific endothelial marker CD31 (red) and DAPI (blue), and the number of CD31-positive microvessels in the intra-tumoral (**I**, ***p < 0.001, n = 5) and peri-tumoral areas (**J**, ****p < 0.0001, n = 5) were quantified, respectively. Data were analyzed by unpaired Student's t-test, one- or two-way ANOVA followed by Bonferroni’s multiple comparison test

Significant bioluminescence intensity indicating distant metastasis was observed in the control group, whereas metastatic tumor masses were rare in the EBA-treated animals (*p* < 0.01, Fig. 6A). Histopathological analysis of lung tissue confirmed that EBA reduced the number of metastatic nodules in lungs (*p* < 0.05, Fig. 6B).

The anti-metastatic effects of EBA were associated with a notable reduction in vimentin expression (*p* < 0.0001, Fig. 6C and Supplementary Fig. S16) in vivo. Kinetic analysis revealed a concentration-dependent impairment of cell migration in the presence of EBA in MDA-MB-231 and 4T1 cells in vitro (*p* < 0.05, Fig. 6D–F and Supplementary Fig. S17). During metastasis, significant increases in MMP-2/-9 concentrations in serum were found in the metastatic allograft mice (**p < 0.01, Fig. 6G–H). This phenomenon was noticeably hampered by EBA administration (##*p* < 0.01, Fig. 6G–H). A microvessel density (MVD) assay revealed that EBA administration resulted in a notable decline in both intra- and peri-tumoral angiogenesis, as evidenced by significant reductions in the number of CD31-positive microvessels in vivo (*p* < 0.001, Fig. 6I–Jand Supplementary Fig. S18).

## Discussion

Drug repositioning involves the identification of novel indications for FDA-approved drugs. Advantages when compared to the development de novo drugs include reduced process failure risk and the ability to leverage established data on safety profiles, tolerability, pharmacodynamics and pharmacokinetics [19]. We report the potent efficacy of EBA, an H1-antihistamine with an exceptional safety profile, as a candidate for drug repurposing due to its potent anti-metastatic potential and suppression of BCSC-like properties. No lethal or toxic effects were previously observed in mice administered at 36 mg/kg/day for up to 80 weeks or in rats administered for 104 weeks. High-dose EBA of 144 mg/kg in rats and 60 mg/kg in dogs for 26 weeks did not reveal any significant toxicological effects. Importantly, there were no mutagenic or reproductive toxicity concerns including fertility, malformations, or perinatal mortality even at doses exceeding 100 mg/kg in rats [20]. Furthermore, clinical pharmacokinetics studies revealed that EBA was well tolerated in patients with impaired renal function or impaired hepatic function [21, 22]. In agreement with our findings, blood biochemical profiles showed no significant changes in biomarkers of kidney and liver function between control- and EBA-treated mice.

The 5-year overall survival rate for TNBC patients with advanced resistance to adjuvant chemotherapy and metastasis is exceedingly low at less than 30% [23]. FAK is an important determinant of tumor angiogenesis, cancer progression and metastasis via its interactions with the extracellular matrix and cytoskeletal reorganization [24]. Docking studies reveal that EBA fits into the tyrosine kinase domain of FAK within the ATP-binding pocket (residues 410–686), blocking phosphorylation at the Y397 and Y576/Y577 residues leading to catalytic dysfunction of FAK.

ALDH1A1 is a major BCSC biomarker associated with significantly lower overall survival in parallel with FAK overexpression in breast cancer patients. High phosphorylation of FAK at the tyrosine Y397 residue in BCSC-like populations is associated with the abnormal overexpression and activation of MEK/ERK and JAK2/STAT3, which enhance aggressive characteristics in TNBC. EBA treatment resulted in a significant reduction in FAK/Src activation coinciding with the attenuation of MEK/ERK and JAK2/STAT3 activation in BCSC-enriched mammospheres. Preclinical studies show that FAK blockade using pharmacological inhibitors or siRNA knockdown reduces BCSC-like properties, including ALDH1 activity, EMT, mammosphere-forming ability and tumor-initiating capacity in breast cancer in vitro and in vivo [25, 26]. EBA-treated TNBC cells also exhibit significant reductions in ALDH1 activity, CD44^high^/CD24^low^ and CD49f^high^/CD24^high^ stem-like subpopulations, leading to the attenuation of spheroid-forming ability. EBA induces apoptosis in BCSC-like populations, as evidenced by a significant increase in cleaved caspase-3 and PARP degradation as well as downregulation of the cancer stem cell markers ALDH1A1, CD49f, Nanog and Sox2 in BCSC-enriched mammospheres.

EBA administration in vivo retarded tumor growth, angiogenesis and lung metastasis in BCSC-enriched allografts. Of particular note, the anti-metastatic and anti-angiogenic properties of EBA was attributable to significant decreases in MMP-2 and MMP-9 serum levels. The matrix metalloproteinases facilitate tumor neovascularization and metastatic spread via the degradation and remodeling of ECM [27, 28]. MMP-2/-9 serum levels were significantly escalated in metastatic allograft mice when compared to normal BALB/c mice of the same age, however, these effects were abolished following EBA treatment in vivo. Notably, MMP-9 concentrations in circulating blood were elevated approximately 50-fold (range 3–5 ng/ml to 200–300 ng/ml) during metastasis, which may contribute to angiogenesis and the spread of tumor cells to distant organs. Accumulating evidence suggests a significant interplay between MMP-9 and CD44 in the progression of metastasis [29]. The complex formation of glycoprotein CD44 and its ligand MMP-9 on the cell surface of breast tumor cells was found to promote ECM degradation, cell migration, invasion and metastasis, suggesting that the MMP-9/CD44 axis is a promising therapeutic target [30]. Co-expression analysis using the TCGA database identified a positive relationship between CD44 and MMP-9 in breast cancer (Supplementary Fig. S19A). Furthermore, Kaplan–Meier analysis showed that patients with high CD44 levels had lower overall survival when MMP-9 was overexpressed (Supplementary Fig. S19B). It is conceivable that the downregulation of CD44 by EBA impacts MMP-9 levels, preventing angiogenesis and metastasis.

**Fig. 7:**
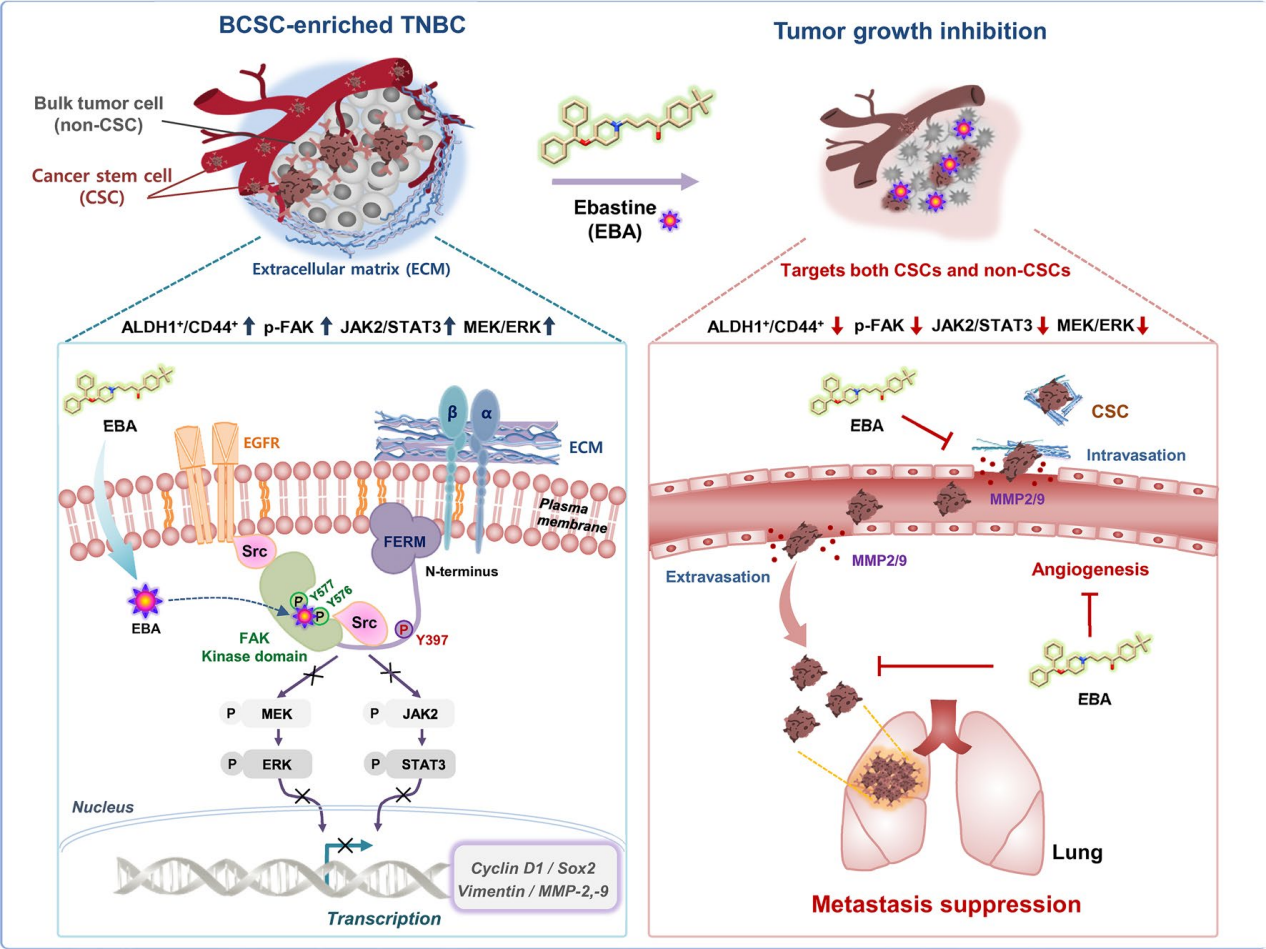
Hypothetical model illustrating the multiple action mechanisms of EBA on tumor growth and metastasis in BCSC-enriched TNBC. TNBC has a heterogeneous nature with higher levels of ALDH1A1, CD44 and CD49f as well as activation of the FAK/ERK/STAT3 pathways. EBA directly binds to the tyrosine kinase domain of FAK and subsequently suppresses phosphorylation of FAK (Y397, Y576/577) in BCSC-enriched TNBC. EBA effectively attenuates FAK/Src-mediated JAK2/STAT3 and MEK/ERK signaling, resulting in significant retardation of tumor growth, angiogenesis and metastasis. The anti-angiogenic and anti-metastatic properties of EBA are associated with STAT3 suppression leading to the downregulation of vimentin and MMP-2/-9 levels. EBA may be suitable for repurposing to simultaneously eliminate rapidly proliferating tumor cells and BCSC-like populations from the tumor bulk

Constitutive activation of STAT3 is essential for the preservation of BCSC-like properties and the acquisition of EMT, with phospho-STAT3 predominantly expressed at the invasive edge in the peritumoral areas of advanced tumors [31, 32]. The transcriptional activity of STAT3 is broadly associated with cancer pathology, and dysfunction in the FAK/STAT3 axis induced by EBA treatment resulted in the impairment of BCSC survival, angiogenesis and tumor propagation. These findings suggest that EBA may be an effective therapeutic repositioning candidate for the simultaneous targeting of multiple survival signaling pathways for the treatment of molecularly heterogeneous TNBC (Fig. 7).

## Materials and methods

### Materials used (details listed in supplementary information)

Reagents, materials and antibodies, cell viability assay, sub-G1 analysis, annexin V/PI assay, aldefluor assay, CD44^high^/CD24^low^ and CD49f^high^/CD24^high^ assay, ALDH-positive and -negative cell sorting and cytological centrifugation, Western blot analysis, immunocytochemistry, molecular docking study, allograft in vivo experiments and bioluminescence imaging, immunohistochemistry and in-situ TUNEL assay, mammosphere formation assays, MMP2/MMP9 ELISA assay, and serum biochemical analysis (ALT, AST and BUN assay), cell migration kinetic assay (wound healing) and public dataset source and bioinformatics analysis.

### Breast cancer cell and normal fibroblast cell culture

The TNBC cell lines MDA-MB-231 (PerkinElmer Inc), BT549, 4T1-Luc (Japanese Collection of Research Bioresources Cell Bank, JCRB) and normal mouse fibroblast line NIH/3T3 (ATCC) were cultured in MEM, DMEM or RPMI 1640 (Gibco, MD) containing 10% fetal bovine serum (FBS), and streptomycin-penicillin (100 U/ml). Cells were incubated at 37 °C in an atmosphere of 5% CO_2_. All cell lines were authenticated by short tandem repeat (STR) profiling by Macrogen Inc (Seoul, South Korea).

### Molecular modeling and docking analysis

The molecular docking studies were conducted using the GalaxySagittarius software. (https://galaxy.seoklab.org/) [33]. After completion of the docking simulation, visualization of the 2D and 3D protein–ligand complexes and predicted binding sites were analyzed using UCSF chimera (https://www.cgl.ucsf.edu/chimera/) and BIOVIA Discovery Studio 2021 (https://discover.3ds.com/discovery-studio-visualizer-download/) [34, 35].

### Allograft in vivo experiments and bioluminescence imaging

All animal procedures were carried out in accordance with guidelines approved by the Korea University Institutional Animal Care and Use Committee (IACUC, KOREA-2021–0070). Five-week-old female BALB/c mice were obtained from the NARA Biotech Animal Center (Seoul, Korea), housed in a pathogen-free environment, and acclimated for 2 weeks prior to the study with free access to food and water. 1 × 10^5^ cells from 4T1 mammospheres were injected into the fourth mammary fat pads of 7-week-old BALB/c female mice. When average tumor volumes reached 50 mm^3^, the animals were randomized into 2 groups (n = 5/each group), and vehicle (DMSO/corn oil, 1:9) or EBA (20 mg/kg·BW/day) was administered intraperitoneally every other day for 34 days, and tumor volumes were measured using a caliper and calculated using the formula V = (Length × Width^2^)/2. After a period of 24 h following the final administration of EBA, the animals were anesthetized and subjected to NightOWL LB983 bioluminescence imaging (BLI) (Berthold Technologies, TN). The procedures were performed as previously described [36].

### Public dataset source and bioinformatics analysis

Gene expression in normal and tumor tissues was analyzed using the publicly-available UCSC Xena (http://xena.ucsc.edu), GENT2 database (http://gent2.appex.kr/gent2/), and METABRIC dataset. Data for survival analyses were downloaded from TCGA and GENT2 databases. Overall survival regression was analyzed with GraphPad Prism 9.0 software after categorization into high- and low-expression groups. Overall survival was analyzed up to 150 months, with *p*-values obtained through the *log-rank* test.

### Statistical analysis

All data were analyzed using GraphPad Prism 9.0 statistical software (San Diego, CA). The results are presented as mean ± SD of at least three independent experiments. Data were analyzed by Student’s *t*-test, and one- or two-way ANOVA as appropriate. Significance between multiple experimental groups was determined using the Bonferroni post-hoc test and defined at *p* < 0.05.

## Supplementary Information

Below is the link to the electronic supplementary material.

Supplementary file1 (DOCX 43 KB)

Supplementary file2 (DOCX 7152 KB)

## Data Availability

Not applicable.
