# Supplementary material for: Ebastine impairs metastatic spread in triple-negative breast cancer by targeting focal adhesion kinase
Source: Cell Mol Life Sci. 2023 Apr 25;80(5):132. doi: 10.1007/s00018-023-04760-5 (PMC10130003; doi:10.1007/s00018-023-04760-5)
Supplement: Supplementary file 1 — Supplementary file1 (DOCX 43 KB) [file 18_2023_4760_MOESM1_ESM.docx]

**Ebastine impairs metastatic spread in triple-negative breast cancer by targeting focal adhesion kinase**

**Supplementary information**

**Supplementary Materials and Methods**

***Reagents and antibodies***

Ebastine was obtained from Selleckchem (Houston, TX). Triton X-100, Tween-20, propidium iodide (PI), corn oil and dimethyl sulfoxide (DMSO) were purchased from Sigma-Aldrich (St. Louis, MO). Phosphatase inhibitor and protease inhibitor cocktail tablets were obtained from Roche Applied Sciences (Penzberg, GER). RNase A was purchased from Invitrogen (Carlsbad, CA). Primary antibodies used targeted the following proteins: ALDH1A1, CD44, CD49f, phospho-Src (Y419), phospho-STAT3 (Y705), Ki-67, and CD31 (Abcam, MA); MEK1/2, phospho-MEK1/2 (S217/221), ERK1/2, EGFR, phospho-EGFR (Y1068), JAK2, phospho-JAK2 (Y1007/1008), FAK, PARP, cleaved-caspase-3, cleaved-caspase-7, cleaved-caspase-8, Sox2, Nanog and vimentin (Cell Signaling, CA); phospho-ERK1/2 (T202/Y204), Src, STAT3, survivin and cyclin D1 (Santa Cruz, CA); phospho-FAK (Y397), phospho-FAK (Y576/577) and GAPDH (Invitrogen, CA).

***Breast cancer cell and normal fibroblast cell culture***

The TNBC cell lines MDA-MB-231 (PerkinElmer Inc), BT549, 4T1-Luc (Japanese Collection of Research Bioresources Cell Bank, JCRB) and mouse normal fibroblast NIH/3T3 (ATCC) were cultured in MEM or RPMI 1640 (Gibco, MD) containing 10% fetal bovine serum (FBS), and streptomycin-penicillin (100 U/ml). Cells were incubated at 37°C in an atmosphere of 5% CO_2_. All cell lines were authenticated by short tandem repeat (STR) profiling by Macrogen Inc (Seoul, South Korea).

***Cell viability assay***

Cell viability was measured using a CellTiter 96* Aqueous One Solution Cell Proliferation Assay [MTS, 3-(4,5-dimethylthiazol-2-yl)-5-(3-carboxymethoxyphenyl)-2-(4-sulfophenyl)-2H-tetrazolium] (Promega, Madison, WI) according to the manufacturer’s instructions. The quantity of formazan product was determined by measuring the absorbance at 490 nm with a Spectramax MAX 190 microplate reader (Molecular Devices, CA).

***Sub-G1 analysis and Annexin V/PI assay***

Cells were harvested and fixed with 95% ethanol containing 0.5% Tween-20 for 24 h, incubated with PI (50 mg/mL) and RNase (50 mg/mL) for 30 min. The annexin V/PI assay was assessed using a FITC-conjugated Annexin V apoptosis detection kit (BD Biosciences) in accordance with the manufacturer’s protocol. Stained cells were analyzed by flow cytometry using a BD LSRFortessa™ X-20 (BD Biosciences, NJ).

***Aldefluor-positivity assay, CD44^high^/CD24^low^ and /CD49f^high^/CD24^high^ staining***

An Aldefluor assay kit (Stemcell Technologies, Vancouver, BC) was used to assess ALDH1 activity, as previously described [1]. As a specific inhibitor of ALDH1, 50 mM diethylamino-benzaldehyde (DEAB) was used as a negative control. For CD44^high^/CD24^low^ and CD49f^high^/CD24^high^ staining, cells were stained with FITC- and PE-conjugated anti-mouse IgG or FITC-conjugated anti-CD24 and PE-conjugated anti-CD44 or CD49f antibodies (BD Biosciences) and analyzed by flow cytometry.

***Immunoblot analysis***

The procedures were performed as previously described [2]. Primary antibody dilutions were: [PARP (1:2000), cleaved-caspase-3 (1:2000), cleaved-caspase-7 (1:2000), cleaved-caspase-8 (1:2000), vimentin (1:2000), Sox2 (1:1000), Oct4 (1:1000), Nanog (1:1000), cyclin D1 (1:2000), survivin (1:2000), EGFR (1:2000), phospho-EGFR (1:1000), MEK1/2 (1:2000), phospho-MEK1/2 (1:2000), ERK1/2 (1:2000), phospho-ERK1/2 (1:2000), JAK2 (1:2000), phospho-JAK2 (1:500), STAT3 (1:2000), phospho-STAT3 (1:1000), ALDH1A1 (1:2000), CD44 (1:2000), CD49f (1:2000), FAK (1:2000), phospho-Src (Y419, 1:500), Src (1:500), phospho-FAK (Y397, 1:1000), phospho-FAK (Y576/577, 1:1000), and GAPDH (1:10000)], followed by incubation with HRP-conjugated rabbit or mouse secondary antibody (1:1000–1:10,000, Bio-Rad). Signal intensity was detected using a Chemiluminescence Kit (Thermo Fisher Scientific, IL) on X-ray film (AGFA Healthcare, Belgium) and quantitated using AlphaEaseFC software (Alpha Innotech, CA).

***Immunocytochemistry***

The procedures were performed as previously described [3]. The cells with primary antibodies FAK (1:100) and phospho-FAK (1:100) in antibody-diluent (Dako, Denmark) were incubated overnight at 4°C, and then incubated with Alexa Fluor®-488 goat anti-rabbit IgG and Texas Red-X Phalloidin (Invitrogen, CA). Cells were mounted with ProLong Gold Antifade Reagent with DAPI (Life Technologies, CA). Images were acquired using a confocal microscope Carl Zeiss LSM 900 (Weimar, GER), and the intensity of the images was analyzed using the intensity profile tool ZEN black v3.0.

***Molecular modeling and docking analysis***

The molecular docking studies were conducted using the GalaxySagittarius software. (https://galaxy.seoklab.org/) [4]. After completion of the docking simulation, visualization of the 2D and 3D protein-ligand complexes and predicted binding sites were analyzed using UCSF chimera (https://www.cgl.ucsf.edu/chimera/) and BIOVIA Discovery Studio 2021 (<https://discover.3ds.com/discovery-studio-visualizer-download/>) [5,6].

***Mammosphere formation assay***

BT549 (1×10^5^/mL) or 4T1 (3×10^4^/mL) cells were plated in ultralow attachment dishes and cultured in HuMEC basal serum free medium (Gibco), supplemented with B27 (1:50, Invitrogen), 20 ng/mL basic fibroblast growth factor (bFGF, Sigma), 20 ng/mL human or mouse epidermal growth factor (EGF, Sigma), 4 μg /mL heparin, 1% antibiotic-antimycotics, and 15 μg/mL gentamycin. The number and volume of the mammospheres were determined under an Olympus CKX53 inverted microscope. Mammosphere volumes were calculated using the formula Volume=4/3*3.14(π)*r^3^ (r: radius).

***Cell sorting and cytological centrifugation***

Aldefluor-positive (ALDH1+) or -negative (ALDH1-) populations in 4T1 mammospheres were sorted by FACS-Melody cell sorters (BD Bioscience), as previously described [7]. The cells were incubated with the primary antibody [ALDH1A1 (1:50) or phospho-FAK (Y397, 1:50)] in antibody diluent at 4°C overnight and then with Alexa Fluor-488 at RT for 2 hrs. Cells were mounted with DAPI, and images were acquired using a confocal microscope.

***Allograft in vivo experiments and bioluminescence imaging***

All animal procedures were carried out in accordance with guidelines approved by the Korea University Institutional Animal Care and Use Committee (IACUC, KOREA-2021-0070). Five-week-old female BALB/c mice were obtained from the NARA Biotech Animal Center (Seoul, Korea), housed in a pathogen-free environment, and acclimated for 2 weeks prior to the study with free access to food and water. 1×10^5^ cells from 4T1 mammospheres were injected into the fourth mammary fat pads of 7-week-old BALB/c female mice. When average tumor volumes reached 50 mm^3^, the animals were randomized into 2 groups (n=5/each group), and vehicle (DMSO/corn oil, 1:9) or EBA (20 mg/kg·BW/day) was administered intraperitoneally every other day for 34 days, and tumor volumes were measured using a caliper and calculated using the formula V=(Length×Width^2^)/2. After a period of 24 h following the final administration of EBA, the animals were anesthetized and subjected to NightOWL LB983 bioluminescence imaging (BLI) (Berthold Technologies, TN). The procedures were performed as previously described [8].

***Serum biochemistry profiles for biomarkers of liver and renal injury***

At sacrifice, blood samples of each animal were collected and serum enzyme activities of aspartate aminotransferase (AST), alanine aminotransferase (ALT), and blood urea nitrogen (BUN) levels were determined with an assay kit following the manufacturer’s protocol (Sigma-Aldrich).

***Immunohistochemistry and in-situ localization of apoptosis (TUNEL)***

The procedures were performed as previously described [9]. Tissue sections with primary antibodies (Ki-67, ALDH1A1, CD44, CD49f, FAK, p-FAK, p-STAT3, p-ERK, vimentin, and CD31) in antibody-diluent were incubated overnight at 4°C. For secondary antibody reactions, the sections were incubated with Alexa Fluor®-594 or -488 conjugated secondary antibodies at RT for 2 hours. TUNEL assays were performed on tissue sections using a TUNEL kit (Roche Applied Sciences) in accordance with the manufacturer’s instructions.

***Wound healing assay***

Cells were seeded to 80~90% confluency in 96-well plates (Essen Biosciences, MI). Wound areas were made with a 96-pin Wound Maker device and washed with PBS to prevent reattachment of dislodged cells. Cells were treated with EBA after wound scratching, and images were automatically acquired and registered every hour up to 48 h and 24 h, respectively, with an IncuCyte™ ZOOM® Kinetic Imaging System (Essen Biosciences). Relative wound density was analyzed using the IncuCyte™ Scratch Wound Cell Migration Software Module.

***MMP-2 and MMP-9 ELISA assay***

MMP-2 and MMP-9 levels in mouse serum were measured using ELISA kits (R&D Systems, Minneapolis, MN), according to the manufacturer’s instructions. The quantity of MMP-2 and MMP-9 was determined by measuring the absorbance at 450 nm with a microplate reader.

***Public dataset source and bioinformatics analysis***

Gene expression in normal and tumor tissues was analyzed using the publicly-available UCSC Xena (http://xena.ucsc.edu), GENT2 database (http://gent2.appex.kr/gent2/), and METABRIC dataset. Data for survival analyses were downloaded from TCGA and GENT2 databases. Overall survival regression was analyzed with GraphPad Prism 9.0 software after categorization into high- and low-expression groups. Overall survival was analyzed up to 150 months, with *p*-values obtained through the *log-rank* test.

***Statistical analysis***

All data were analyzed using GraphPad Prism 9.0 statistical software (San Diego, CA). The results are presented as mean ± SD of at least three independent experiments. Data were analyzed by Student’s *t*-test, and one- or two-way ANOVA as appropriate. Significance between multiple experimental groups was determined using the Bonferroni post-hoc test and defined at *p* < 0.05.

**References**

1. Kim JY, Cho Y, Oh E, Lee N, An H, Sung D, Cho TM, Seo JH (2016) Disulfiram targets cancer stem-like properties and the HER2/Akt signaling pathway in HER2-positive breast cancer. Cancer Lett 379:39-48. <https://doi.org/10.1016/j.canlet.2016.05.026>

2. Oh E, Kim JY, Cho Y, An H, Lee N, Jo H, Ban C, Seo JH (2016) Overexpression of angiotensin II type 1 receptor in breast cancer cells induces epithelial-mesenchymal transition and promotes tumor growth and angiogenesis. Biochim Biophys Acta 1863:1071-1081. <https://doi.org/10.1016/j.bbamcr.2016.03.010>

3. Oh E, Kim JY, Sung D, Cho Y, Lee N, An H, Kim YJ, Cho TM, Seo JH (2017) Inhibition of ubiquitin-specific protease 34 (USP34) induces epithelial-mesenchymal transition and promotes stemness in mammary epithelial cells. Cell Signal 36:230-239. <https://doi.org/10.1016/j.cellsig.2017.05.009>

4. Yang J, Kwon S, Bae SH, Park KM, Yoon C, Lee JH, Seok C (2020) GalaxySagittarius: Structure- and Similarity-Based Prediction of Protein Targets for Druglike Compounds. J Chem Inf Model 60:3246-3254. <https://doi.org/10.1021/acs.jcim.0c00104>

5. Abdel-Hamid MK, McCluskey A (2014) In silico docking, molecular dynamics and binding energy insights into the bolinaquinone-clathrin terminal domain binding site. Molecules 19:6609-6622. <https://doi.org/10.3390/molecules19056609>

6. Haque A, Baig GA, Alshawli AS, Sait KHW, Hafeez BB, Tripathi MK, Alghamdi BS, Mohammed Ali HSH, Rasool M (2022) Interaction Analysis of MRP1 with Anticancer Drugs Used in Ovarian Cancer: In Silico Approach. Life (Basel) 12. <https://doi.org/10.3390/life12030383>

7. Oh E, Kim YJ, An H, Sung D, Cho TM, Farrand L, Jang S, Seo JH, Kim JY (2018) Flubendazole elicits anti-metastatic effects in triple-negative breast cancer via STAT3 inhibition. Int J Cancer 143:1978-1993. <https://doi.org/10.1002/ijc.31585>

8. Kim JY, Cho TM, Park JM, Park S, Park M, Nam KD, Ko D, Seo J, Kim S, Jung E, Farrand L, Nguyen CT, Hoang VH, Thanh La M, Ann J, Nam G, Park HJ, Lee J, Kim YJ, Seo JH (2022) A novel HSP90 inhibitor SL-145 suppresses metastatic triple-negative breast cancer without triggering the heat shock response. Oncogene 41:3289-3297. <https://doi.org/10.1038/s41388-022-02269-y>

9. An H, Kim JY, Lee N, Cho Y, Oh E, Seo JH (2015) Salinomycin possesses anti-tumor activity and inhibits breast cancer stem-like cells via an apoptosis-independent pathway. Biochem Biophys Res Commun 466:696-703. <https://doi.org/10.1016/j.bbrc.2015.09.108>
