# Supplementary material for: Ebastine impairs metastatic spread in triple-negative breast cancer by targeting focal adhesion kinase
Source: Cell Mol Life Sci. 2023 Apr 25;80(5):132. doi: 10.1007/s00018-023-04760-5 (PMC10130003; doi:10.1007/s00018-023-04760-5)
Supplement: Supplementary file 2 — Supplementary file2 (DOCX 7152 KB) [file 18_2023_4760_MOESM2_ESM.docx]

**Ebastine impairs metastatic spread in triple-negative breast cancer by targeting focal adhesion kinase**

**Supplementary information**

**Supplementary Figures and Legends (Fig. S1-S19)**

***Supplementary Fig. S1***

**
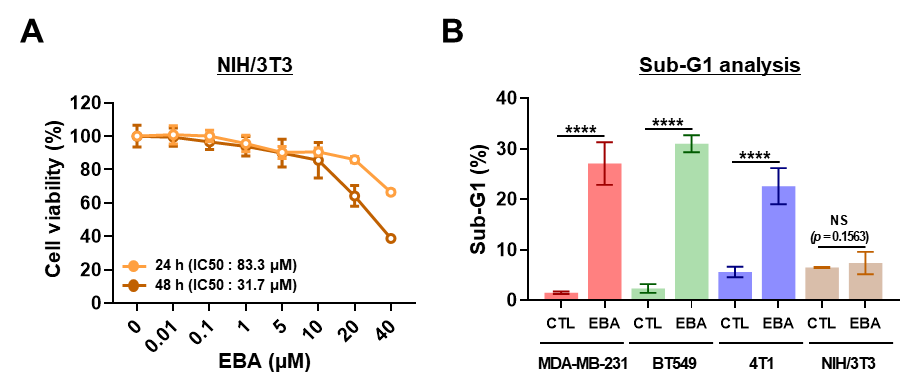
**

**Supplementary Fig. S1. EBA exhibit less cytotoxicity toward normal mouse fibroblast NIH/3T3 cells. A** Cells were treated with the indicated concentrations (0.01-40 μM) of EBA for 24 and 48 h. Cell viability and IC_50_ values were determined by MTS assay (n=5). **B** Comparison of effect of EBA on cell death in TNBC and normal fibroblast cells. MDA-MB-231, BT549, 4T1 and NIH/3T3 cells were treated with EBA (20 μM, 48 h), and the percentages of cells in the sub-G1 phase were quantified (****p<0.0001; NS, not significant).

***Supplementary Fig. S2***


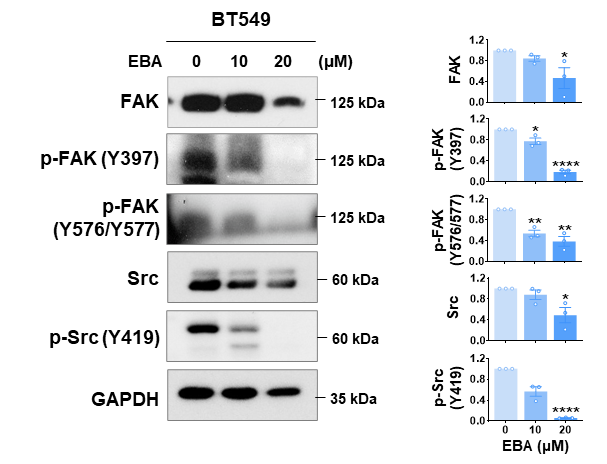


**Supplementary Fig. S2. Influence of EBA on the expression and phosphorylation of FAK and Src in BT549 cells.** Immunoblot analyses of FAK, p-FAK (Y397), p-FAK (Y576/577), Src and p-Src (Y419) following exposure to EBA (0-20 μM, 48 h) in BT549 cells. Quantitative graphs represent the ratio of each protein/GAPDH in the presence or absence of EBA (*p<0.05, n=3).

***Supplementary Fig. S3***

**
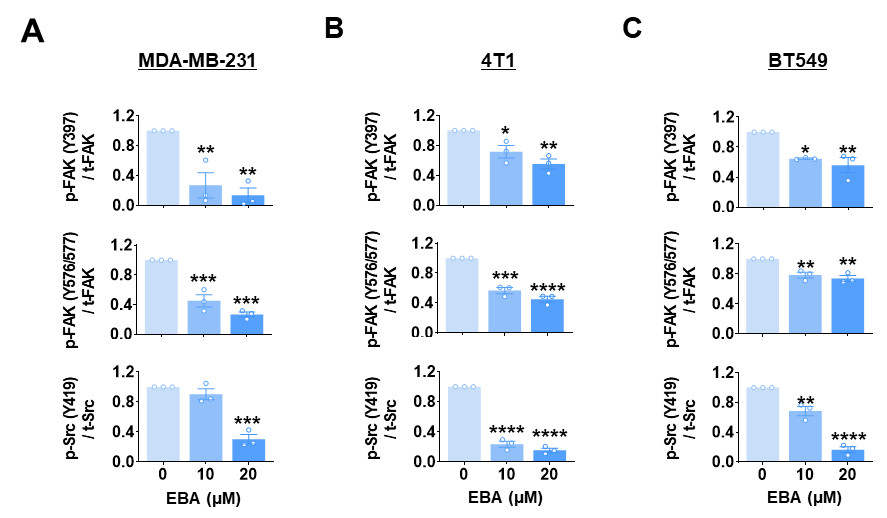
**

**Supplementary Fig. S3. Influence of EBA on phosphorylation of FAK and Src, corresponding to Figure 2E and 2F in the main text.** **A-C** Quantitative graphs for the ratios of p-FAK (Y397)/total FAK, p-FAK (Y576/577)/total FAK and p-Src (Y419)/total Src in MDA-MB-231 (**A**), 4T1 (**B**) and BT549 (**C**) cells following exposure to EBA (10-20 μM, 48 h) (**p*<0.05, n=3).

***Supplementary Fig. S4***

**
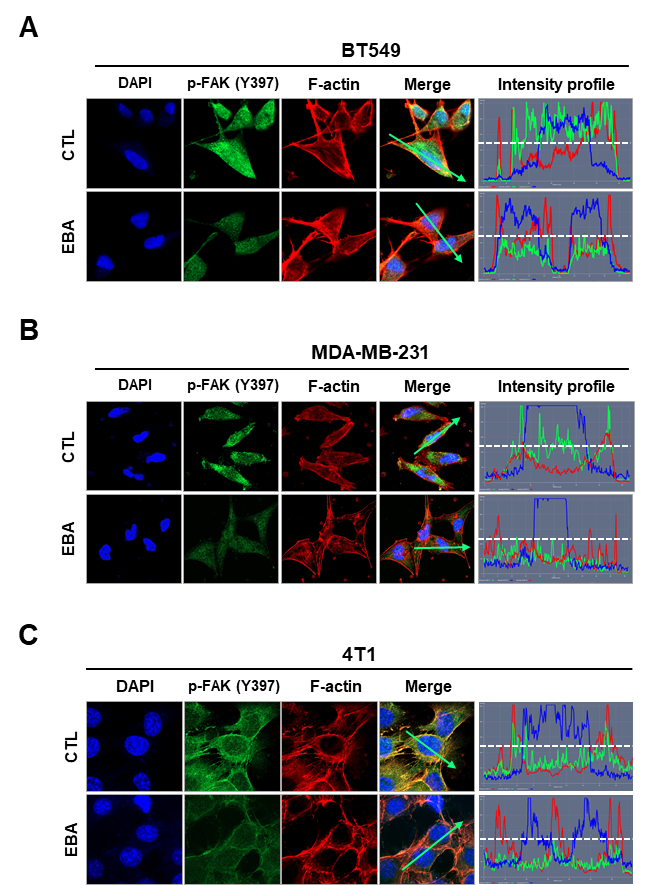
**

**Supplementary Fig. S4. Effect of EBA on expression of phospho-FAK (Y397) in BT549, MDA-MB-231 and 4T1 cells,** **corresponding to Fig. 2G in the main text. A-B** Immunofluorescence analysis of p-FAK (Y397, green) and F-actin (red) with DAPI (blue) in BT549 and MDA-MB-231 cells after treatment with EBA (10 μM, 48 h). **C** Immunostaining of 4T1 cells was performed after exposure to EBA (10 μM) for 24 h. The intensity profiles represent p-FAK with green signal fluorescence and were analyzed using a histogram tool in the Carl Zeiss software. The horizontal line (white dotted line) indicates 125 intensity units (y-axis on the left; range: 0-250 units).

***Supplementary Fig. S5***

**
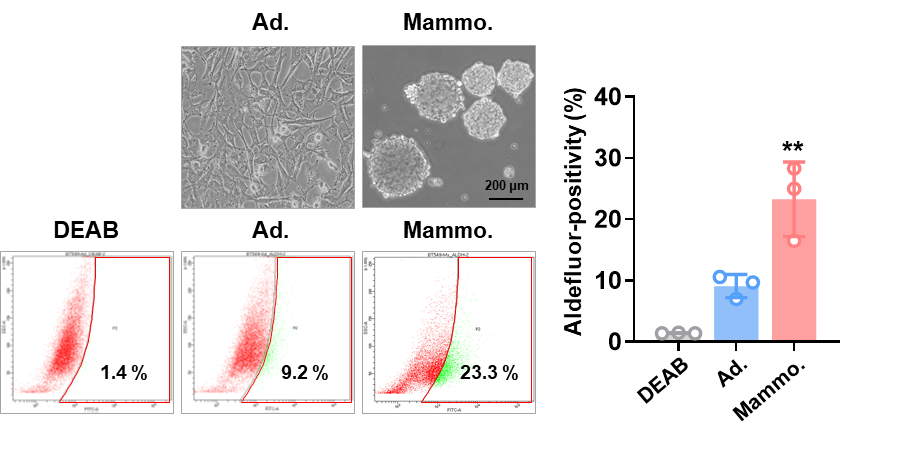
**

**Supplementary Fig. S5. Representative phase-contrast images and ALDH1 activity between adherent BT549 cells and mammospheres, corresponding to Fig. 3C in the main text.** BT549 cells (1×10^5^ cells/mL) were cultured in normal culture medium or anchorage-independent serum-free 3D spheroid medium for 4 days and ALDH1 activity was assessed by flow cytometry. The percentage of Aldefluor-positive cells was quantified (***p*<0.01, n=3). Results are presented as mean values ± SD of three independent experiments. Data were analyzed by Student’s t test.

***Supplementary Fig. S6***


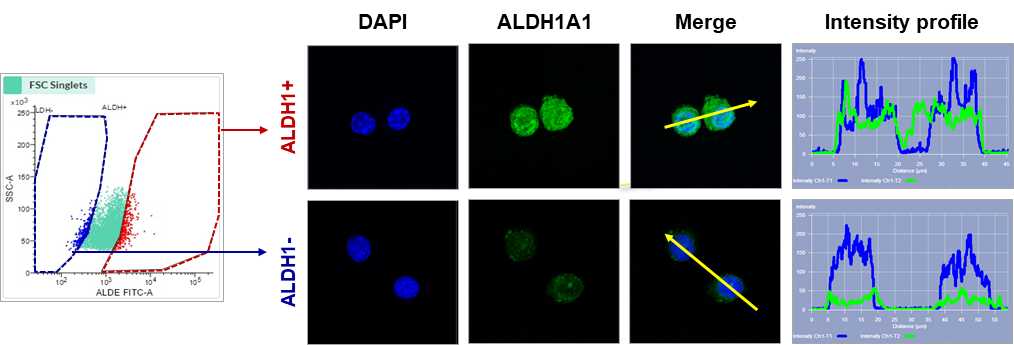


**Supplementary Fig. S6. Immunocytochemical analysis for ALDH1A1 expression in ALDH1 positive (+) and negative (-) 4T1 cells, corresponding to Fig. 3F in the main text.** ALDH1 (+) or ALDH1 (-) cells were sorted from dissociated 4T1 mammospheres and immunostained for ALDH1A1 (green) with DAPI (blue). The fluorescence intensity was analyzed using the intensity profiling tool in the confocal microscopy software (original magnification: ×500).

***Supplementary Fig. S7***

**
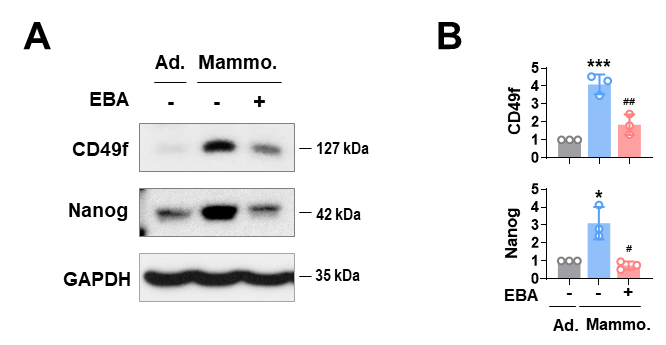
**

**Supplementary Fig. S7. EBA targets BCSC-like properties. A-B** 4T1 cells (3×10^4^ cells/mL) were cultured in normal culture medium or anchorage-independent serum-free 3D spheroid medium for 4 days. **A** Changes in the expression of CD49f and Nanog following exposure to EBA (5 μM) for 4 days in 4T1 mammosphere culture. GAPDH was used as an internal loading control. **B** Quantitative graphs of protein expression levels are shown (**p*<0.05, Ad. vs Mammo.; #*p*<0.05, DMSO control vs EBA treatment in mammospheres, n=3).

***Supplementary Fig. S8***

**
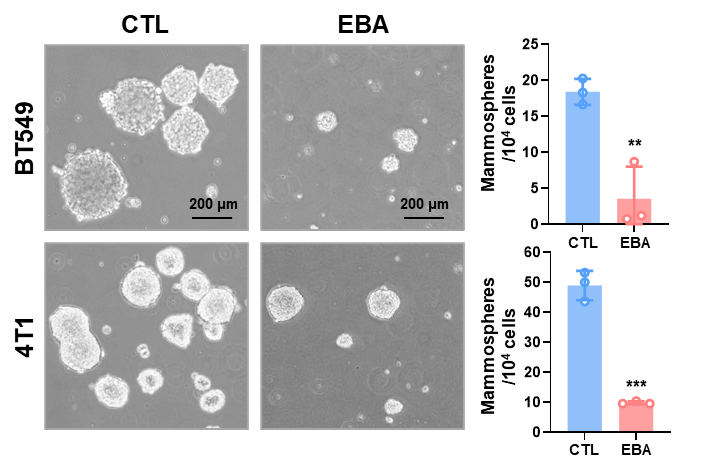
**

**Supplementary Fig. S8. Effect of EBA on mammosphere formation, corresponding to Fig. 3J in the main text.** BT549 (1×10^5^ cells/mL) and 4T1 cells (3×10^4^ cells/mL) were plated in ultralow attachment dishes, cultured in the presence or absence of EBA (5 μM) for 4 days, and the number of mammospheres was quantified by optical microscopy (***p*<0.01, n=3). Results are presented as mean values ± SD of at least three independent experiments and were analyzed by Student’s t test.

***Supplementary Fig. S9***

**
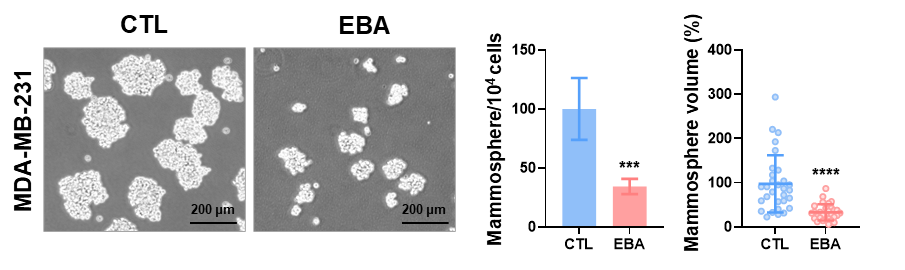
**

**Supplementary Fig. S9. Influence of EBA on mammosphere formation *in vitro*.** MDA-MB-231 cells (1×10^5^ cells/mL) were plated in ultralow attachment dishes, cultured in the presence or absence of EBA (5 μM) for 7 days, and the number and volume of mammospheres was quantified by optical microscopy (****p*<0.001, n=3).

***Supplementary Fig. S10***

**
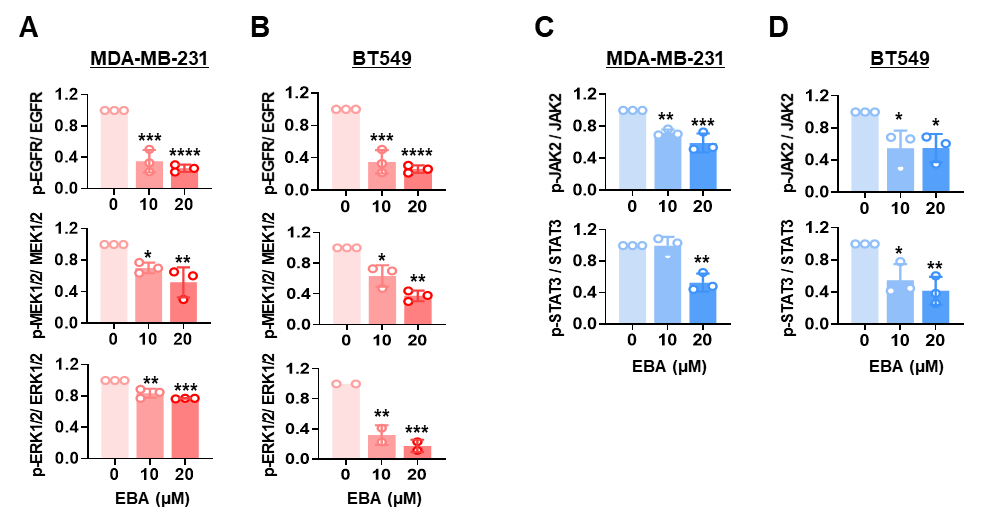
**

**Supplementary Fig. S10. Effects of EBA on phosphorylation of EGFR, MEK, ERK, JAK2 and STAT3, corresponding to Figure 4A-D in the main text.** **A-B** Quantitative graphs of the ratios of p-EGFR (Y1068)/total EGFR, p-MEK1/2 (S217/221)/total MEK1/2 and p-ERK1/2 (T202/Y204)/total ERK1/2 in MDA-MB-231 (**A**) and BT549 (**B**) cells following exposure to EBA (10-20 μM, 48 h) (**p*<0.05, n=3). **C-D** The ratios of p-JAK2 (Y1008/1007)/total JAK2, p-STAT3 (Y705)/total STAT3 in MDA-MB-231 (**C**) and BT549 (**D**) cells following exposure to EBA (10-20 μM, 48 h) (**p*<0.05, n=3).

***Supplementary Fig. S11***

**
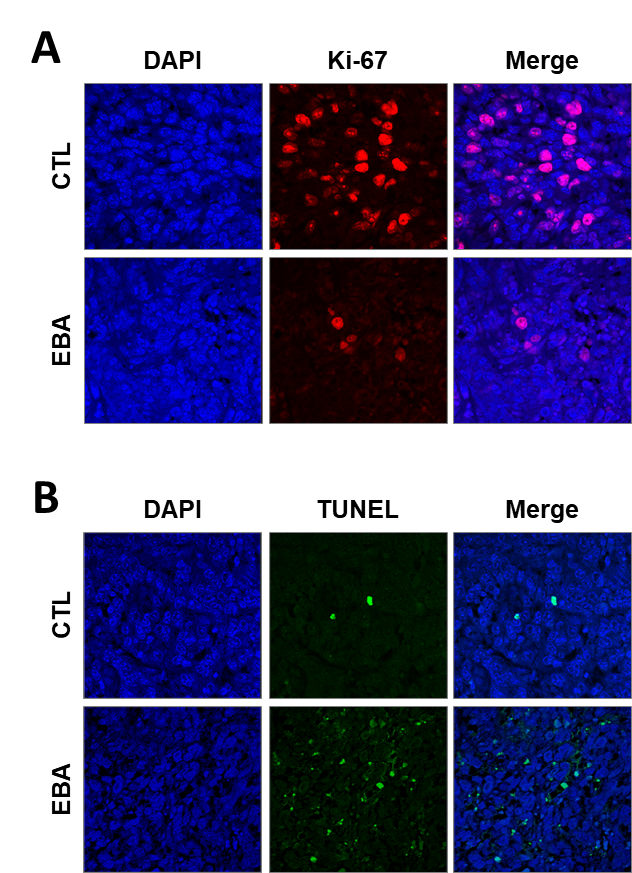
**

**Supplementary Fig. S11** **Effects of EBA on Ki-67 expression and apoptosis *in vivo*, corresponding to Fig. 5F and 5G in the main text. A** Tissue sections were immunostained for Ki-67 (red) with DAPI (blue). **B** EBA-induced apoptosis was determined by TUNEL assay. All images were taken with a confocal microscope (original magnification: ×500).

***Supplementary Fig. S12***

**
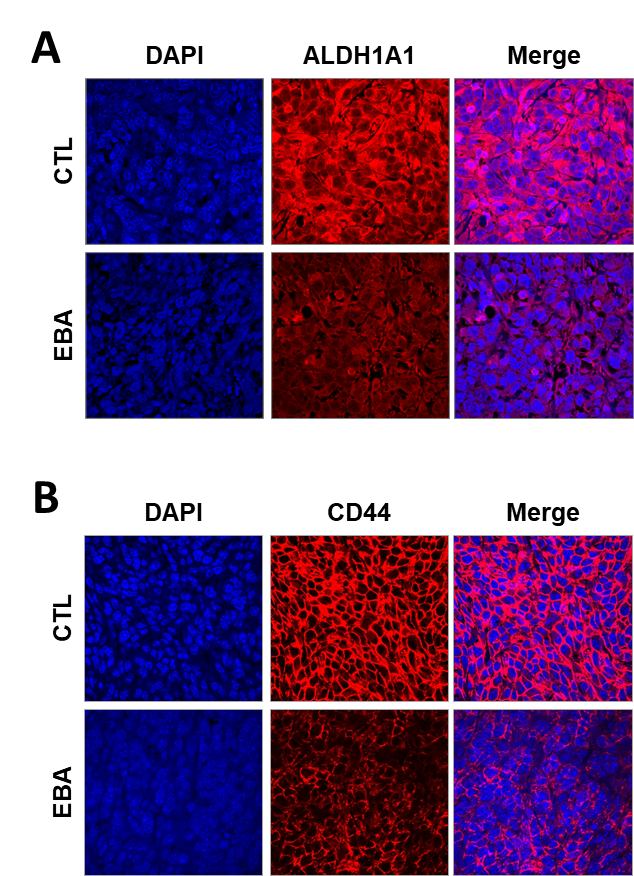
**

**Supplementary Fig. S12. Influence of EBA on ALDH1A1 and CD44 expression *in vivo*, corresponding to Fig. 5H and 5I in the main text. A-B** Immunofluorescence analysis of ALDH1A1 and CD44 in allografts derived from 4T1 mammospheres. Tumor tissue sections were immunostained with ALDH1A1 (**A**, red) and CD44 (**B**, red) antibodies, and counterstained with DAPI (blue). Original magnification: ×500.

***Supplementary Fig. S13***


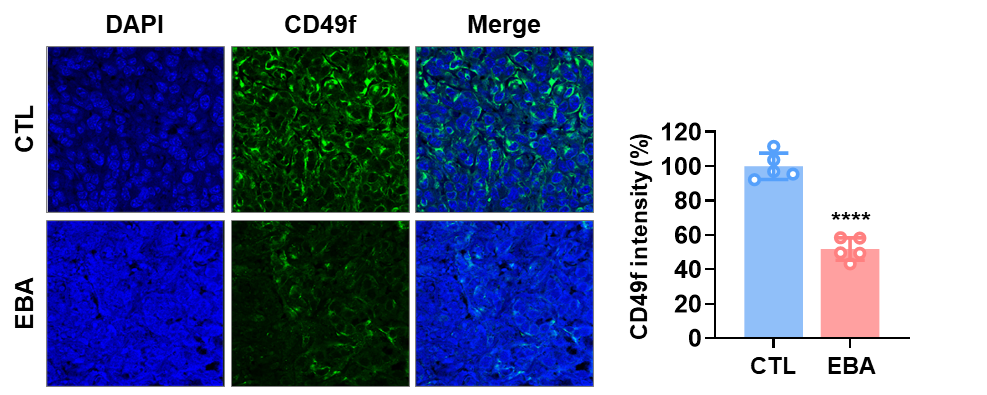


**Supplementary Fig. S13. Influence of EBA on CD49f expression in allograft tumors *in vivo*.** Tumor tissue sections were immunostained with CD49f (green) with DAPI (blue), and fluorescence intensity was quantified (*****p*<0.0001, n=5). The results are presented as mean ± SD and data were analyzed by unpaired Student’s t-test.

***Supplementary Fig. S14***

**
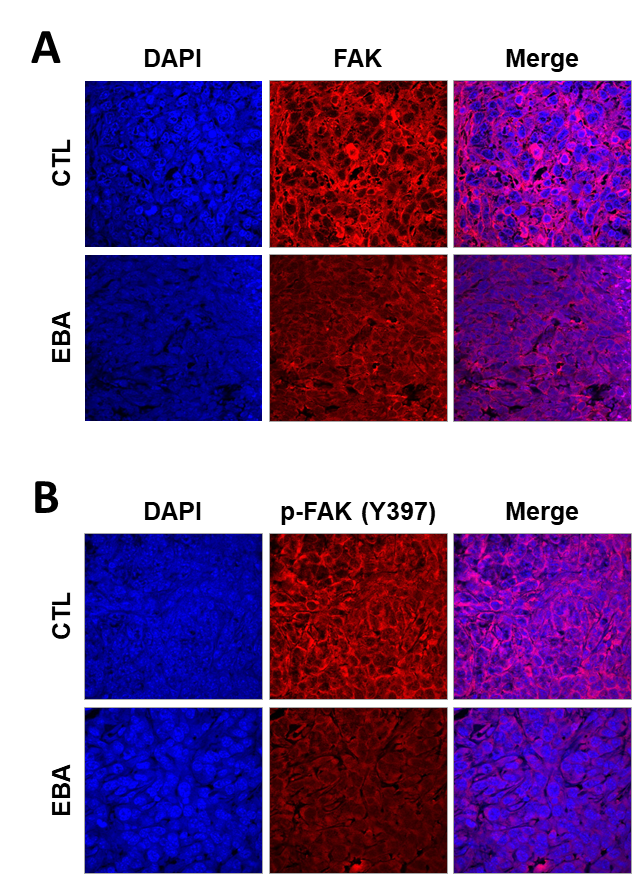
**

**Supplementary Fig. S14. EBA markedly downregulates the expression of FAK and phospho-FAK (Y397) *in vivo,* corresponding to Fig. 5J and 5K in the main text.** Tissue sections were immunostained with FAK (**A**, red) and p-FAK (**B**, red), and counterstained with DAPI (blue). Original magnification: × 500.

***Supplementary Fig. S15***

***
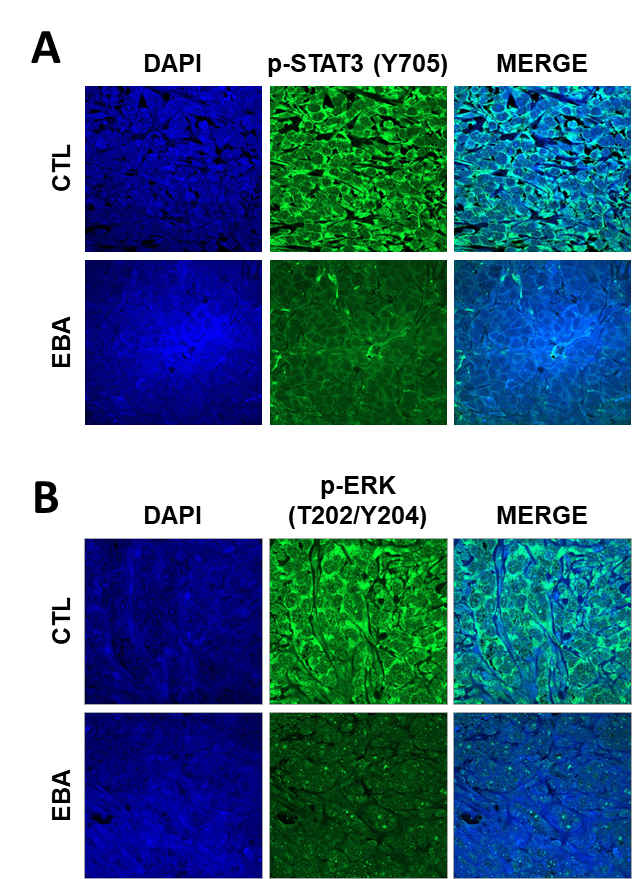
***

**Supplementary Fig. S15. Impact of EBA on the expression of phospho-STAT3 and phospho-ERK in 4T1 mammosphere allograft tumors*,* corresponding to Fig. 5L and 5M in the main text.** Tumor sections were immunostained for p-STAT3 (**A**, green), and p-ERK (**B**, green) with DAPI (blue). Original magnification: × 500.

***Supplementary Fig. S16***


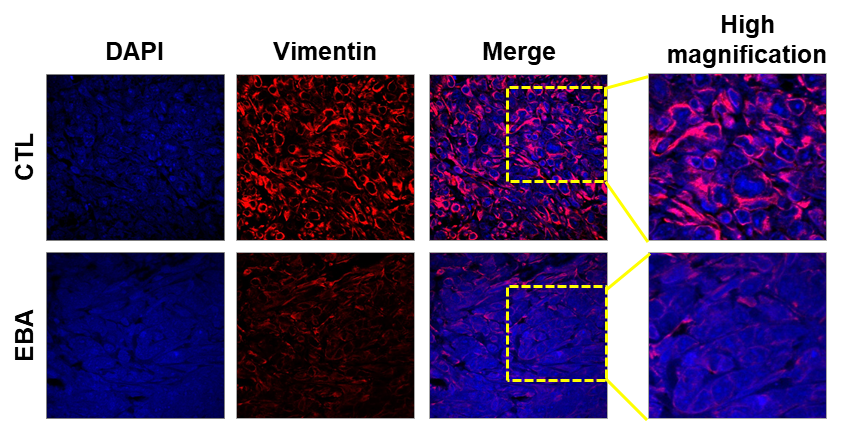


**Supplementary Fig. S16. Change in the expression of vimentin following exposure to EBA in 4T1 mammosphere allografts*,* corresponding to Fig. 6C in the main text.** Tumor tissue sections were immunostained for vimentin (red) and were counterstained with DAPI (blue). Original magnification: ×500.

***Supplementary Fig. S17***

**
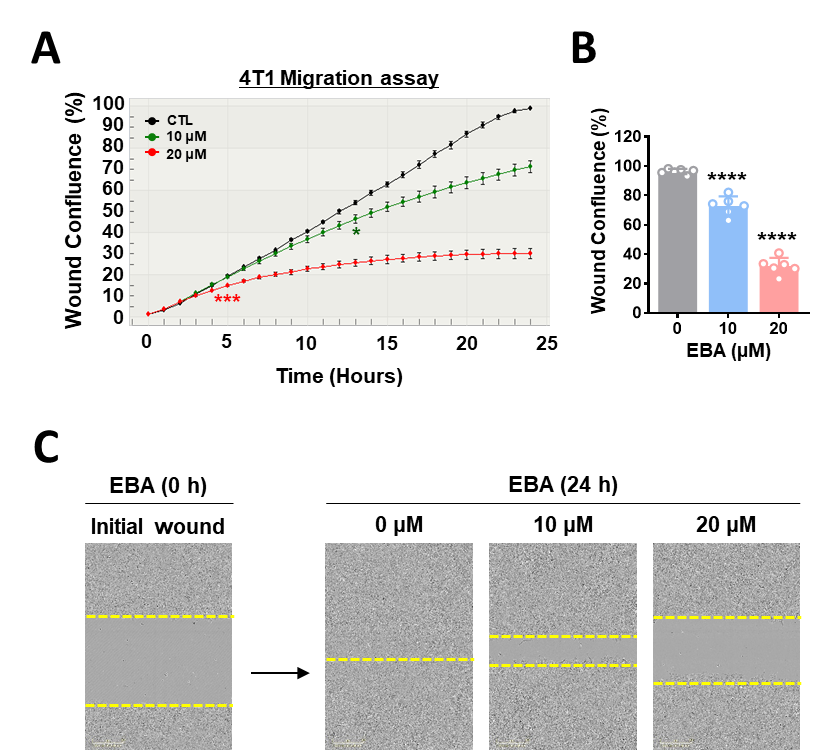
**

**Supplementary Fig. S17. Effect of EBA on cell migration in 4T1 cells, corresponding to Fig. 6D-6F in the main text. A-C** 4T1 cells were treated with EBA (0-20 µM) for 24 h. **A** The kinetic analysis of cell migration was determined using the IncuCyte™ Live-Cell Imaging System and quantified for the indicated time duration (**p*<0.05, n=6). **B** The quantitative graph represents the relative wound density (%) in 4T1 cells at 24 h (*****p*<0.001, n=6). **C** Representative images of wound closure by cell migration at 0 and 24 h after EBA treatment (0-20 μM). The yellow dotted line indicates the edge of the scratched wound. Data were analyzed by one- or two-way ANOVA followed by Bonferroni’s multiple comparison test.

***Supplementary Fig. S18***

**
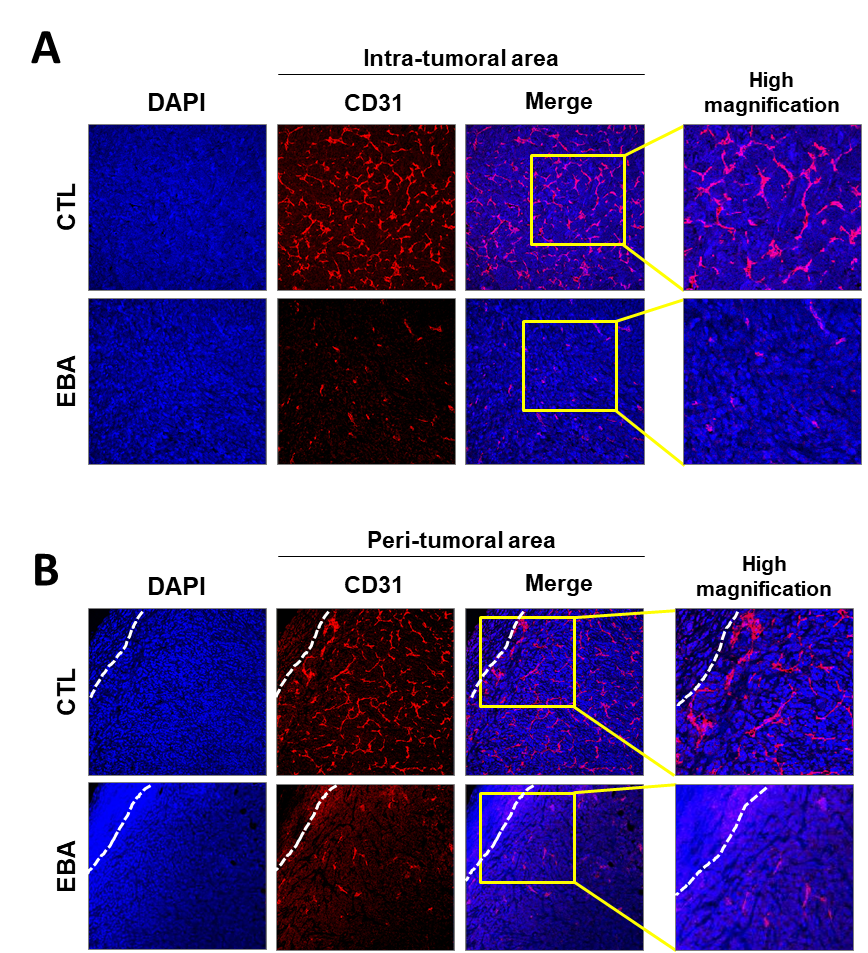
**

**Supplementary Fig. S18. Influence of EBA on tumor angiogenesis *in vivo*, corresponding to Fig. 6I and 6J in the main text. A-B** Tumor tissues were immunostained with a specific endothelial marker CD31 (red) and nuclei were stained with DAPI (blue). Significant reductions in CD31-positive microvessels in both intra-tumoral (**A**) and peri-tumoral (**B**) areas were observed in the EBA-treated group. Fluorescent images of CD31 with DAPI are shown at high magnification (original magnification: ×200).

***Supplementary Fig. S19***

**
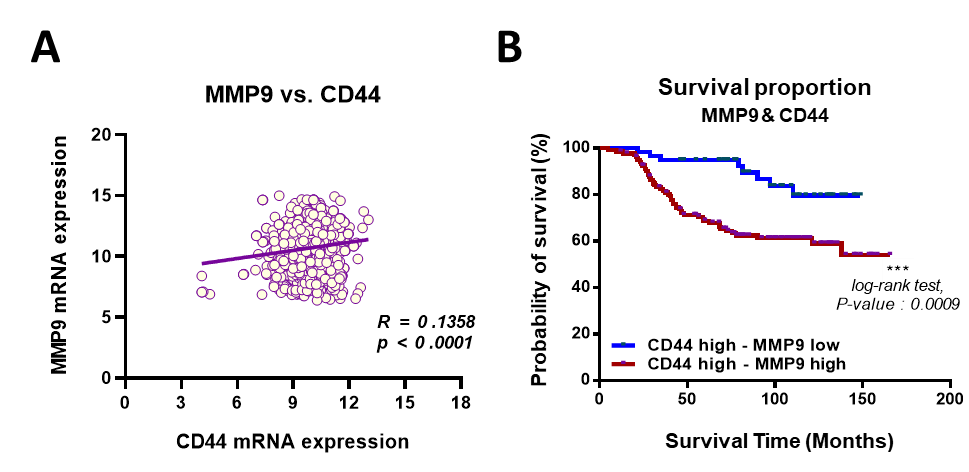
**

**Supplementary Fig. S19. Significant correlation between MMP-9 and CD44 expression in breast cancer patients. A** Correlation of mRNA levels between MMP-9 and CD44 was analyzed (*****p*<0.0001, n=2164). **B** Kaplan-Meier curves represent overall survival of breast cancer patients according to MMP-9 and CD44 expression. With the high expression of MMP-9 and CD44, shorter survival was observed [*p*=0.0009, CD44-high/MMP9-low (n=57) and CD44-high/MMP9-high (n=135)]. Statistical significance and Kaplan-Meier survival analysis were determined by *log-rank* (Mantel-Cox) test.
